# Molecular dynamics study of Cl^−^ permeation through cystic fibrosis transmembrane conductance regulator (CFTR)

**DOI:** 10.1007/s00018-022-04621-7

**Published:** 2023-01-24

**Authors:** Zhi Wei Zeng, Paul Linsdell, Régis Pomès

**Affiliations:** 1 Molecular Medicine, Hospital for Sick Children, 686 Bay Street, Toronto, ON M5G 0A4 Canada; 2 Department of Biochemistry, University of Toronto, Toronto, ON M5S 1A8 Canada; 3 Department of Physiology and Biophysics, Dalhousie University, PO Box 15000, Halifax, NS B3H 1X5 Canada

**Keywords:** Membrane protein, ATP-binding cassette, Protein dynamics, Molecular mechanism, Pore hydration, Ion solvation

## Abstract

The recent elucidation of atomistic structures of Cl^−^ channel CFTR provides opportunities for understanding the molecular basis of cystic fibrosis. Despite having been activated through phosphorylation and provided with ATP ligands, several near-atomistic cryo-EM structures of CFTR are in a closed state, as inferred from the lack of a continuous passage through a hydrophobic bottleneck region located in the extracellular portion of the pore. Here, we present repeated, microsecond-long molecular dynamics simulations of human CFTR solvated in a lipid bilayer and aqueous NaCl. At equilibrium, Cl^−^ ions enter the channel through a lateral intracellular portal and bind to two distinct cationic sites inside the channel pore but do not traverse the narrow, de-wetted bottleneck. Simulations conducted in the presence of a strong hyperpolarizing electric field led to spontaneous Cl^−^ translocation events through the bottleneck region of the channel, suggesting that the protein relaxed to a functionally open state. Conformational changes of small magnitude involving transmembrane helices 1 and 6 preceded ion permeation through diverging exit routes at the extracellular end of the pore. The pore bottleneck undergoes wetting prior to Cl^−^ translocation, suggesting that it acts as a hydrophobic gate. Although permeating Cl^−^ ions remain mostly hydrated, partial dehydration occurs at the binding sites and in the bottleneck. The observed Cl^−^ pathway is largely consistent with the loci of mutations that alter channel conductance, anion binding, and ion selectivity, supporting the model of the open state of CFTR obtained in the present study.

**Supplementary Information:**

The online version contains supplementary material available at 10.1007/s00018-022-04621-7.

## Introduction

Cystic fibrosis transmembrane conductance regulator (CFTR) is a chloride channel found in cells of epithelial tissues [1–4]. Vital for the secretory function of various tissues, this ion channel regulates the transport of ions and water onto the apical surface of epithelia lining the airways, sweat glands, and the lumen of exocrine organs [5–10]. Loss-of-function mutations in the gene encoding CFTR result in the lethal disease cystic fibrosis (CF). Because many organs rely on secretion for proper function, CF patients experience a wide range of symptoms such as malnutrition and infertility. The lethal consequence of CF is respiratory failure due to recurring infections, as CF patients’ lungs have an impaired ability to secrete mucus needed to rid pathogens [11]. Due to its profound clinical significance, CFTR has attracted much research attention aiming to unravel the mechanism of its function and dysfunction.

CFTR is a member of the ATP-binding cassette (ABC) transporter superfamily, specifically the ABCC exporter subfamily [1, 12]. Like closely related ABC proteins, particularly those of the type IV structural subfamily [13], CFTR consists of two transmembrane domains (TMDs), which create a transport pathway across the membrane, and two nucleotide-binding domains (NBDs), which bind and hydrolyse ATP to facilitate substrate translocation through the TMDs [14]. CFTR also contains a disordered regulatory domain (R-domain) that can be phosphorylated. Remarkably, CFTR is the only member of the ABC proteins known to function as an ion channel. Whereas typical ABC transporters utilize energy from ATP-hydrolysis to actively transport substrates against a transmembrane concentration gradient, CFTR couples its gating cycles to ATP binding and hydrolysis, allowing controlled, rapid movement of ions along the electrochemical gradient [15, 16]. Gating is also regulated by protein kinase A (PKA), which is required to activate the CFTR channel by binding and/or phosphorylating its R-domain [17, 18].

Elucidating both open and closed state structures of the CFTR ion channel, as well as the molecular mechanisms governing transitions between these states, is crucial for understanding CFTR function and dysfunction. A number of near-atomic resolution structures of full-length CFTR have been determined by cryo-electron microscopy (cryo-EM) [19–24]. The structures were determined in two distinct functional states: the dephosphorylated, ATP-unbound state (inactive or inter-burst closed state); and the phosphorylated, ATP-bound state (putative open state). The protein used in structural determination of the putative open state contains the mutation E1371Q, which abolishes ATPase activity and is known to stabilize the open state [25]. More recently, structures of potentiator-bound and corrector-bound human CFTR in the putative open state were also determined, although they do not differ significantly from the one without these ligands [23, 24]. In the inter-burst closed state structure, the cavity of the channel is open to the intracellular space but not to the extracellular space, comparable to the “inward-facing” (IF) conformation of closely related ABC transporters [15, 19]. The dephosphorylated R-domain is thought to be at least partially positioned between two TMD-NBD pairs, effectively preventing the two NBDs from dimerizing [19, 20]. ATP-binding-induced NBD dimerization is required for functional conformational changes to occur in the TMDs [25–27]. Indeed, in the putative open state structure, disengagement of the R-domain, possibly due to phosphorylation, allows the NBDs to bind ATP and dimerize; the TMDs also adopt a different conformation compared to the closed-state structure [21, 22]. In closely related ABC exporters, ATP binding induces the “outward-facing” (OF) conformation characterized by a wide opening on the extracellular end and closed-off intracellular access [15, 28]. In this IF-to-OF, “alternating-access” transport mechanism, the transporters ensure that the transmembrane cavity is never accessible from both sides of the membrane simultaneously [15, 29]. In contrast, the cavity of CFTR in the “OF conformation” (i.e. when the NBDs are dimerized) is still accessible from the intracellular space. Because CFTR functions as an ion channel, its ion-conducting state necessarily requires openings on both sides of the membrane. Due to this feature, CFTR has also been described as a “broken transporter” that bears a single gate at the extracellular end of the TMDs [21, 30].

In electrophysiological measurements, applying PKA and Mg-ATP to CFTR results in the opening of the channel with a single-channel conductance of 6–10 pS for Cl^−^ ions [20, 31]. However, the new structures do not explain how CFTR facilitates Cl^−^ permeation. This is because none of the structures of phosphorylated CFTR in the ATP-bound, NBD-dimerized state show an open passage across the TM region that is sufficiently wide to accommodate water or Cl^−^ ions [21, 22]. Accordingly, a heuristic computational method predicted that the cryo-EM models of both zebrafish and human CFTR contain a dry (or “dewetted”) hydrophobic bottleneck and are therefore closed [32], suggesting that these structures do not represent the ion-conducting state observed under experimental and physiological conditions.

The fact that the cryo-EM-derived structure should be open but is apparently closed raises the question of the conformational changes required to reach the open state. It has been speculated that this NBD-dimerized-yet-closed conformation may be stabilized by the non-native environment in detergent micelles and may require additional transmembrane helical movements to reach the open state [21]. Indeed, a plausible reason why the channel is in a closed state is that its structure was determined under temperature and solvent conditions that are too different from those found in the native lipid membrane. In support of that hypothesis, previous studies have shown that the relaxation of ion channels from a closed state to an open state can occur spontaneously in repeated molecular dynamics (MD) simulations in which the channel is embedded in a lipid bilayer, at room temperature, under conditions favouring the open state [33, 34]. To this date, MD simulations of the phosphorylated, ATP-bound zebrafish CFTR channel have shown that this closed state conformation with dimerized NBDs is stable over hundreds of nanoseconds in a lipid bilayer [35, 36]. Nevertheless, in one of these studies, the hydrophobic bottleneck of CFTR was transiently wide enough to fit Cl^−^ ions, and enhanced sampling simulations were used to predict the pathway and the energetics of Cl^−^ translocation [36]. However, that study stopped short of depicting the open state of the channel, the conformational changes governing channel opening, or the molecular mechanism of Cl^−^ permeation. The aim of the present study is to provide a detailed structural model of the open state of human CFTR and to characterize the mechanism of ion permeation in that structure.

To this end, we conducted repeated, microsecond-long MD simulations of ATP-bound, NBD-dimerized human CFTR starting from the apparently occluded cryo-EM structure, successively in the absence and in the presence of voltage. In previous MD studies, applied voltage was shown to promote activation and deactivation transitions of a potassium channel [37] and to induce the conductive state of an HCN channel starting from a structure of the deactivated state [38]. In another study, applied voltage facilitated the transition from a dry (dewetted) state to a hydrated (wetted) state in a model hydrophobic channel, a prerequisite for ion permeation [39]. In the present study, the hydrophobic bottleneck of the CFTR channel remained largely de-wetted and no ion permeation events occurred in the absence of transmembrane voltage. However, two out of ten simulations conducted in presence of a hyperpolarizing voltage led to spontaneous displacements of pore-lining TM helices, wetting of the bottleneck, and subsequent translocation of Cl^−^ through the entire length of the pore, providing a plausible model of the open state of the CFTR channel derived from recent cryo-EM structures.

## Methods

### Molecular system

We performed MD simulations of human CFTR in its ATP-bound, NBD-dimerized state embedded in a POPC bilayer and solvated in 150 mM NaCl aqueous solution. The structural model of phosphorylated, ATP-bound human E1371Q CFTR (PDB: 6MSM) was used for all simulations [22]. Residues that were missing in the PDB structure include the loops connecting each TMD-NBD pair (410–434, 1174–1201), the R-region (638–844), the extracellular loop between TM7 and TM8 (890–899; also known as the extracellular loop 4), and the segment at the C-terminal end of NBD2 (1452–1489). These missing segments were not modelled in this study, resulting in our structural model consisting of five peptide chains. All chains were acetylated at the N termini and amidated at the C termini into primary amides. Mg-ATP moieties from the original PDB structure were found at the NBD interface and were retained for the simulations. All other species present in the PDB structure were removed if they were not known regions of CFTR or Mg-ATP. These species include cholesterol, phospholipids, and the helix at the TMD-NBD interface believed to be part of the R-domain.

Before embedding the CFTR protein into lipids, the PPM server of Orientations of Proteins in Membranes (OPM) database was used to determine the starting position of the lipid bilayer [82]. The model for CFTR with Mg-ATP bound was then embedded in a POPC bilayer and solvated in water with 150 mM NaCl using the CHARMM-GUI server [83, 84], with no water or ions allowed inside the channel pore. The hexagonal periodic unit cell configuration was chosen with starting dimensions: *a* = *b* = 11 nm, *c* = 18.5 nm, *α* = *β* = 90°, and *γ* = 120°. This procedure resulted in a total of 255 POPC molecules added around the protein within a periodic image.

### Simulation setup and protocol

All MD simulations were conducted using GROMACS 2018 or 2019 [85]. The CHARMM36 forcefield was used for protein, lipids, ions, ATP, together with the TIP3P water model [86–89]. Simulations were run in the *NpT* ensemble (*T* = 300 K, *p* = 1 atm) at 2 fs integration timesteps. Constant temperature was maintained using the Nosé–Hoover thermostat (*τ*_T_ = 0.5) [90, 91]; constant pressure was maintained using the Parrinello-Rahman barostat (*τ*_p_ = 2.0) [92, 93]. Semi-isotropic pressure coupling was used, with isothermal compressibility set to 4.5 × 10^–5^ bar^−1^ both in the *xy*-plane and along the *z*-axis. Nonbonded interactions were calculated using Verlet neighbour lists [94, 95]. Lennard–Jones interactions were cut off at 1.2 nm and a force-based switching function with a range of 1.0 nm was used. The particle-mesh Ewald (PME) method was used to compute electrostatic interactions with a real-space cut-off of 1.2 nm [96, 97]. The LINCS algorithm was used to constrain covalent bonds involving H atoms [98].

The simulation system was first subjected to steepest descent energy minimization until maximum force dropped below 1000 kJ/mol/nm. Random velocities were generated at the beginning of the *NpT* equilibration phase, which was conducted in three 10-ns stages, successively with protein heavy atoms, protein backbone atoms, and protein Cα atoms restrained (with force constants of 1000 kJ/mol/nm^2^ in *x*, *y*, and *z* directions). After the *NpT*-equilibration phase, the simulation was divided into two groups for the production run. In one group, the simulation was conducted in *NpT* ensemble at the same temperature and pressure without applied restraints. In the other group, a uniform electric field of magnitude 32 mV/nm was added along the *z*-axis, normal to the lipid bilayer and pointing towards the intracellular side (negative along *z*-axis). At this electric field strength, the perturbation on the hydration of Cl^−^ ions is minimal [99]. During the production runs, the *z*-dimension of the simulation box (i.e. parameter *c* of unit cell) equilibrated to around 16.5 nm, which gave rise to a transmembrane voltage of approximately − 500 mV. This approach has been used to generate a membrane potential in MD simulations without having to maintain asymmetric ion concentration on both sides of the membrane [100, 101]. In both sets of simulations, new random velocities were generated at the beginning of production runs. Ten 1-μs-long simulation runs were produced from each set of conditions.

### Analysis of simulation data

All molecular snapshots of CFTR from production run trajectories were aligned to the first frame (i.e. the configuration at the end of the equilibration phase) through minimization of RMSD of Cα atoms of TMDs.

All the visualizations and renderings of molecular representations of proteogenic components and ions were done with VMD [102]. The origin of the *z*-axis was chosen to be the position of the Cα atom of T338. To identify ions located inside the channel pore, a simple cylindrical method was used to define the boundaries. The Cα atoms of pore-lining helices (TM1, 2, 6, 8, 11, and 12) located within − 40 < *z* < 20 Å were selected. The centre-of-mass of these Cα atoms in the *xy*-plane was used as the centre of the cylinder. The average distance in the *xy*-plane from the centre to these Cα atoms was used as the radius.

Analyses of the ions inside the channel (axial position and solvation shell), the structure of pore-lining helices, the hydration of the bottleneck region (including solvent-accessible surface area) were done with homemade Tcl scripts aided by VMD and/or python scripts aided by MDTraj [102, 103]. In particular, to analyze ion binding and solvation, a molecule or residue is considered to form a direct contact with a Cl^−^ ion if it contains any atom within 3 Å of the ion. This cut-off criterion is based on radial pair distribution functions computed using VMD (Fig. S11). According to the distribution functions, the closest contacting atoms are most likely hydrogen atoms. The hydropathy and channel path radius analyses were aided by MOLE2 [104].

## Results

### Overview of the channel vestibule

**Fig. 1:**
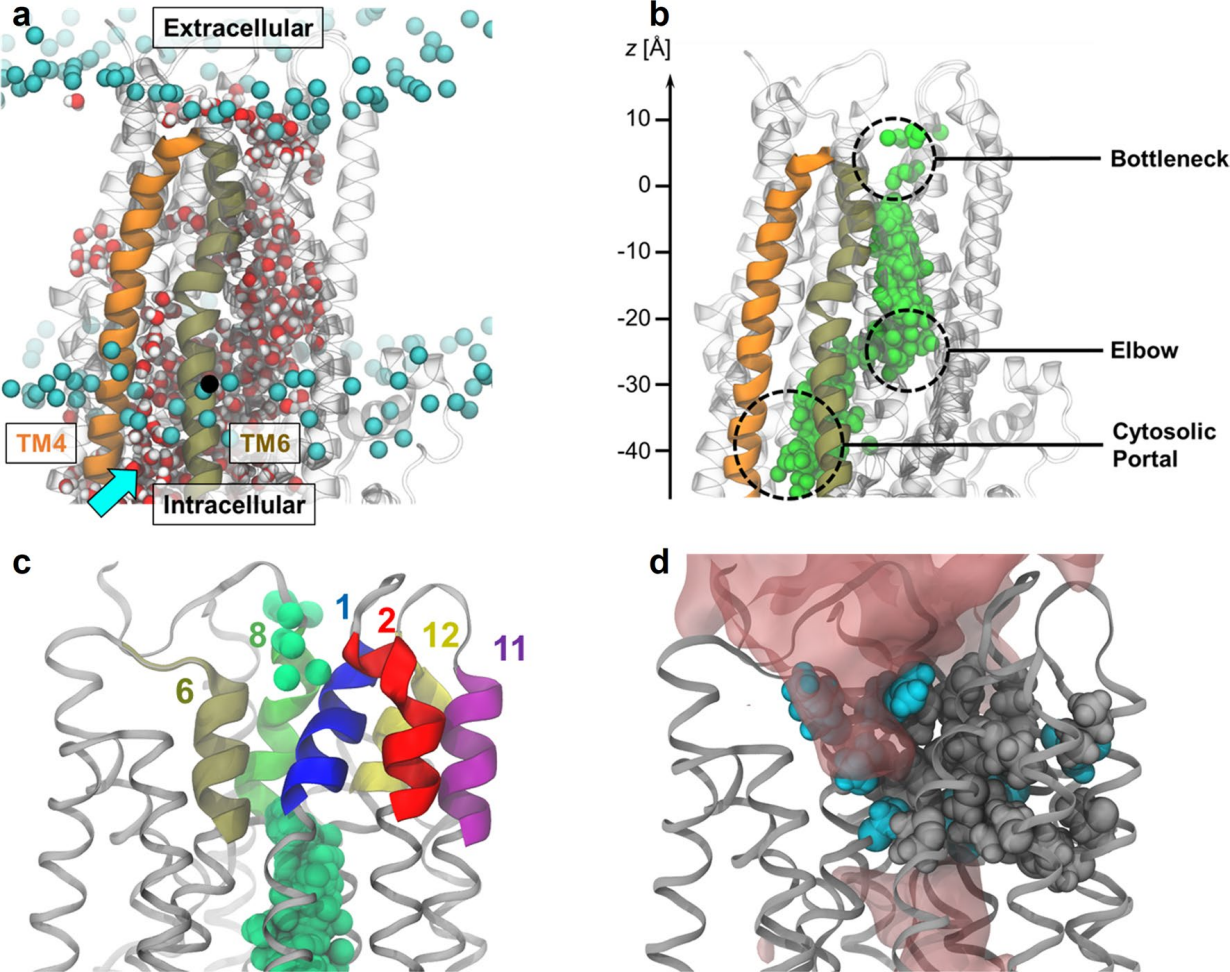
Overview of the CFTR channel pore. **a** Simulation snapshot showing the water-filled channel cavity (inner vestibule). The inner vestibule is accessible to water from the intracellular space through the “cytosolic portal” (cyan arrow) formed by TM helices 4 and 6. Phosphorus atoms (cyan spheres) of POPC head groups indicate membrane position. Bulk water and lipid tails are omitted for clarity. **b** Spatial view of occupancy of Cl^−^ ions (green spheres) inside the channel cavity from many time steps overlaid. **c** Cartoon representation of TM helix segments lining the bottleneck region of the pore together with Cl^−^ ions drawn from multiple time steps. **d** The bottleneck region of the pore is lined by many hydrophobic side chains (grey spheres), disfavouring the presence of water (red surfaces). Selected hydrophilic/charged residues (cyan) are also shown

In all simulations, water filled the transmembrane channel cavity through a lateral intracellular opening formed by cytosolic extensions of transmembrane (TM) helices 4 and 6 and known as the “cytosolic portal” from structural and functional data (Fig. 1a) [19, 20, 40]. Chloride ions entered from the intracellular space through the same opening, whereas sodium ions did not enter the cavity (Fig. 1b). This observation suggests that the cytosolic portal imparts selectivity for anions over cations, possibly due to the abundance of positively charged residues at this location [21, 41].

**Fig. 2:**
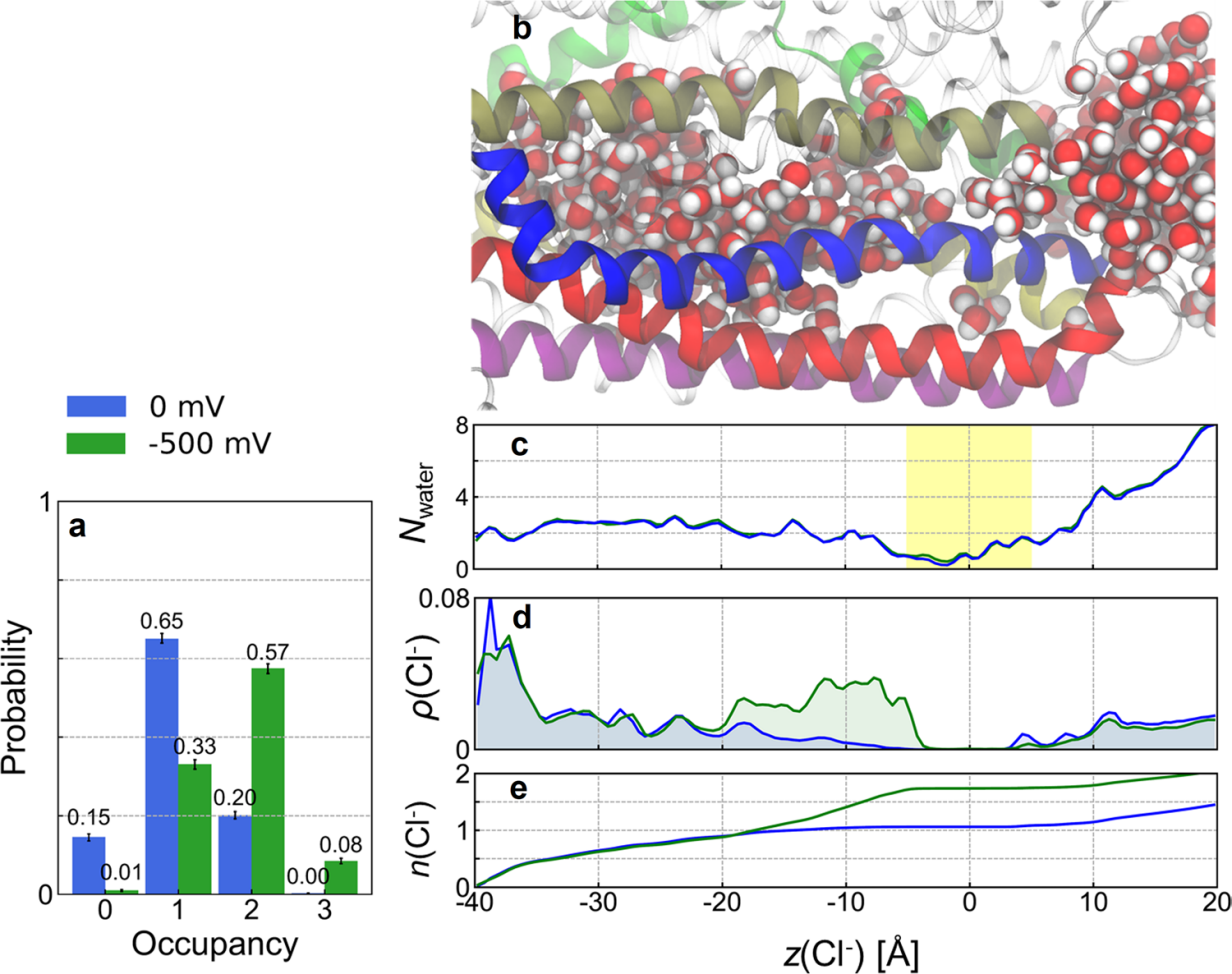
Effect of transmembrane voltage on water and Cl^−^ occupancy of the CFTR channel. **a** Probability distribution of the number of Cl^−^ ions occupying the inner vestibule (blue) without and (green) with applied voltage. Error bars represent bootstrapped 99% confidence intervals. **b** Snapshot of the CFTR channel filled with water molecules used as positional reference for (**c**–**e**). **c** Average number density of water molecules (*N*_water_) in the channel vestibule computed along the *z*-axis. The bottleneck region is highlighted in yellow shading. **d** Average population density of Cl^−^ ions, *ρ*(Cl^−^), inside the channel pore (blue) without and (green) with − 500 mV transmembrane voltage. The Cl^−^ occupancy of the region − 20 < *z* < 0 Å is enhanced by voltage. **e** Cumulative population densities of Cl^−^ ions, *n*(Cl^−^), inside the channel pore computed by integrating *ρ*(Cl^−^) from (**d**)

Unlike most ion channels, in which the ion permeation pathway tends to be linear and symmetrically shaped, CFTR presents a curved passage to Cl^−^ ions. Two elbow-shaped turns create a short, laterally oriented segment at *z* ≊ − 25 Å (Fig. 1b). An apparent barrier opposing Cl^−^ passage is found at the extracellular end of the transmembrane domains (− 5 Å < *z* < 5 Å), suggesting the presence of an energetic or steric bottleneck (Figs. 1b, 2d). Lined by TM helices 1, 2, 6, 8, 11, and 12, this bottleneck region is rich in hydrophobic residues and is the most dewetted region of the channel pore (Figs. 1c, d, 2b, c). In the absence of voltage, the ions reached as far as *z* = − 5 Å inside the cavity, but Cl^−^ permeation did not occur. To induce Cl^−^ permeation, we simulated the same system in the presence of a uniform electric field along the membrane normal. This electric field creates an effective membrane potential of − 500 mV without affecting the structural integrity of the TMDs (Fig. S1). In particular, the conformation of TM8 was conserved over microseconds of simulation time, with the RMSD of its extracellular segment reaching ~ 2 Å after 1 μs relative to the PDB structure, irrespective of voltage or opening status (Fig. S1). The presence of the electric field increased the average ionic occupancy of the inner vestibule from 1.05 to 1.71 (Fig. 2a, e). This extra Cl^−^ density occupies the region of the inner vestibule closest to the bottleneck (Fig. 2d, e: − 20 Å < *z* < − 5 Å). Despite the high transmembrane voltage, the apparent bottleneck for Cl^−^ persisted; however, Cl^−^ translocation events occurred in two out of ten 1-μs-long simulations, as described in detail below (see Results section “Cl^−^ permeation through the bottleneck region”).

### Cl^−^ binding sites in the inner vestibule

**Fig. 3:**
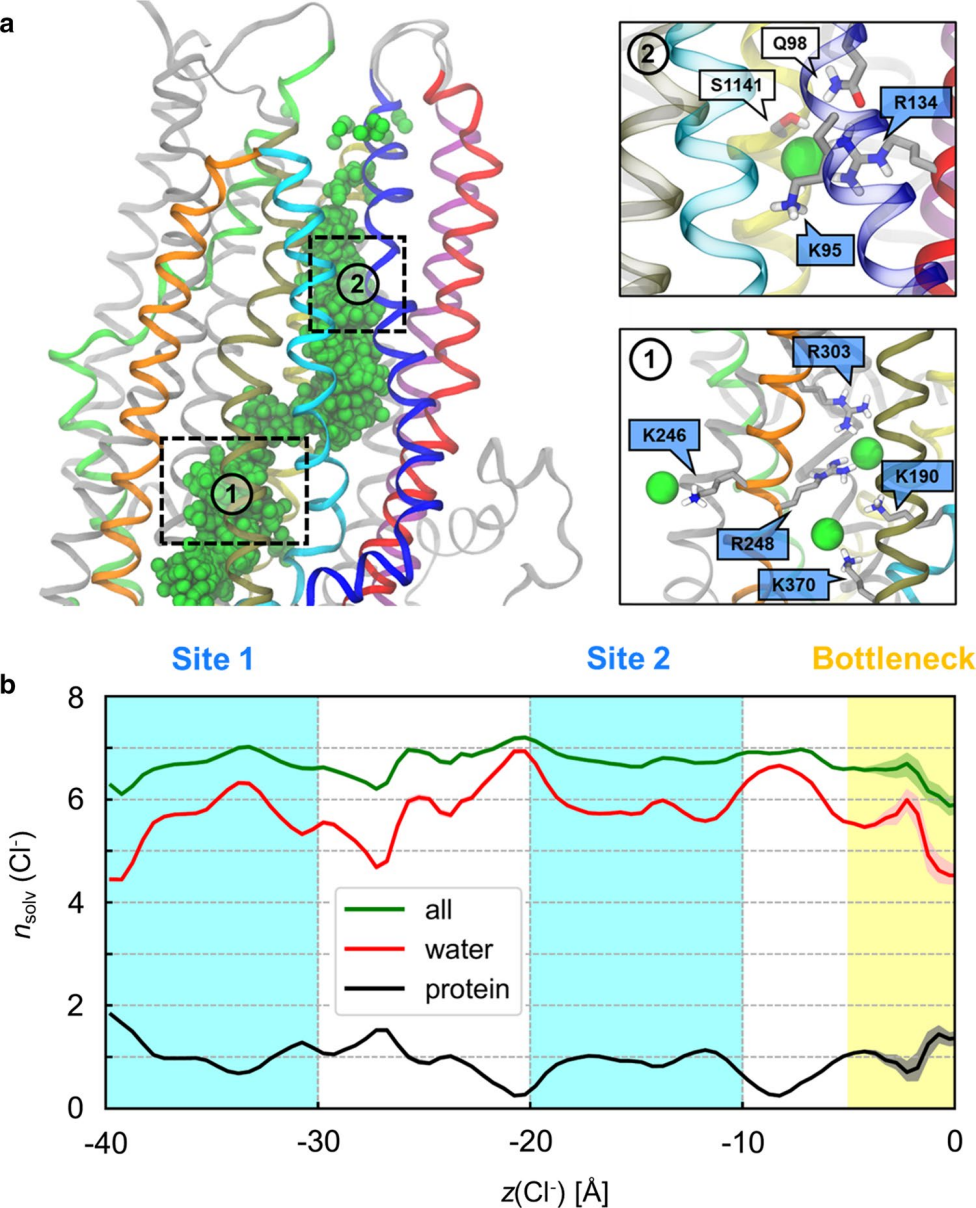
Chloride solvation in the inner vestibule. **a** Left: overview of the Cl^−^ permeation pathway with overlaid ions (green spheres) from simulations in the presence of voltage with TM helices 1 (blue), 2 (red), 3 (cyan), 4 (orange), 6 (tan), 8 (lime), 11 (purple), and 12 (yellow). The locations of Cl^−^ binding sites 1 and 2 are indicated in dashed boxes. Right: close-up views of binding sites 1 and 2. Amino acid sidechains in direct contact with Cl^−^ ions are shown. The labels of positively charged residues, including K246 and K370 from the cytosolic portal, are shaded in indigo. **b** Coordination of Cl^−^ ions inside the channel cavity from simulations with voltage. The average numbers of protein residues (black) and water molecules (red) in the first solvation shell of Cl^−^ ions (*n*_solv_) along the intracellular pathway are shown together with their sum (green). Shading represents 99% bootstrapped confidence intervals. Error bars are largest in the bottleneck region, which is least visited by Cl^−^ ions

The analysis of the average Cl^−^ distribution in the pore (Fig. 2d) suggests the presence of multiple ion binding sites in the inner vestibule, where protein residues directly contacted Cl^−^ ions through the first solvation shell. In particular, two major binding sites were identified where Cl^−^ ions resided on average for 20–50 ns and up to 400 ns (see Supplementary Methods and Fig. S2). The first site is located near the cytosolic portal and consists primarily of residues K190, R248, and R303 (Fig. 3a). The second binding site is located 10–15 Å above the elbow region and consists primarily of K95, R134, and Q98 (Fig. 3a). In the absence of an electric field, site 2 is the farthest location reached by Cl^−^ ions. The presence of an electric field increased the occupancy of site 2 from 0.14 to 0.50 (Fig. 2d, e). Between binding sites 1 and 2, Cl^−^ bound more transiently to two other sites: one involving R352 and W356, with some contribution from R303; and the other involving primarily R1097 (Fig. S3). Additionally, there is another auxiliary binding site located adjacent to site 1 formed by K1041 and R1048 from TM10 (Fig. S4). Regardless of whether an electric field was present, the sidechains of these residues appeared to orient towards the opening between TM4 and TM6 and bind Cl^−^ ions. Because R1048 is located at the interface between the intracellular space and the inner vestibule, it can bind intracellular Cl^−^ ions approaching the hypothetical cytosolic portal formed by TM10 and TM12 (Fig. S4). However, no Cl^−^ ions entered the inner vestibule through TM10-TM12 portal, contrary to results obtained for zebrafish CFTR (see “Discussion”).

Chloride binding resulted in time periods during which the Cl^−^ ion stayed relatively stationary along the channel pathway, even in the presence of voltage (Figs. S5, S6). Although multiple protein residues can bind Cl^−^ ion at either site, on average only one protein residue bound Cl^−^ ions in sites 1 and 2, respectively. Chloride ions remained well hydrated irrespective of the electric field (Fig. 3b).

### Cl^−^ permeation through the bottleneck region

**Fig. 4:**
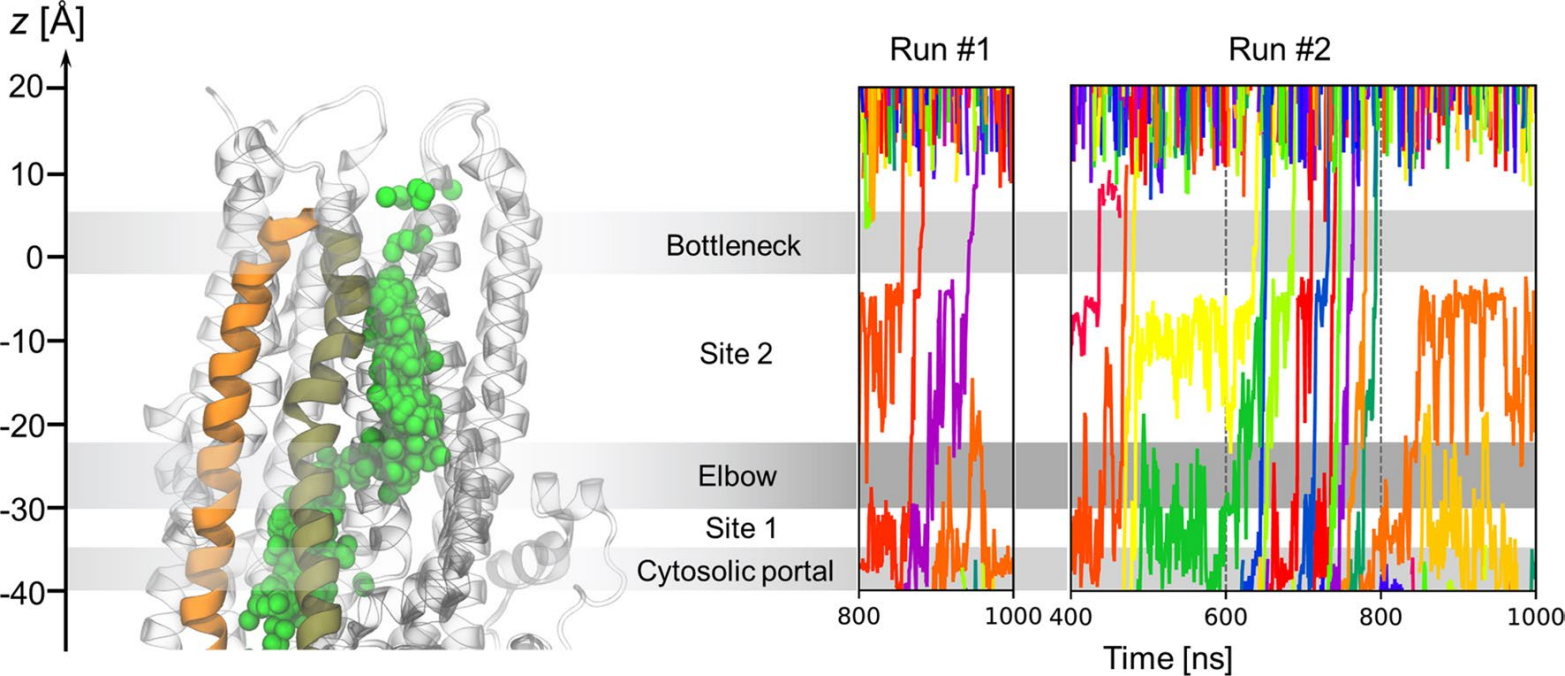
Spontaneous permeation of Cl^−^ ions in the CFTR channel under applied voltage. Left: overview of the Cl^−^ permeation pathway with overlaid ions (green spheres) from simulations in presence of voltage highlighting TM helices 4 (orange) and 6 (tan). Right: time series of axial Cl^−^ ion positions from the two simulation runs in which permeation occurred (− 500 mV). Ion permeation events are indicated by traces passing through the bottleneck region. Individual Cl^−^ ions are distinguished by colour for clarity

A total of 17 Cl^−^ translocation events occurred in runs #1 and #2 (Fig. 4; Fig. S6; Table S1). These permeation events were closely spaced in time and occurred in three apparent bursts, suggesting interconversions between conductive and non-conductive states. Run #1 contained one such burst involving 3 Cl^−^ permeation events (800 < *t* < 1000 ns) and run #2 contained two bursts (400 < *t* < 500 ns and 600 < *t* < 800 ns) involving 3 and 11 Cl^−^ permeation events, respectively.

**Fig. 5:**
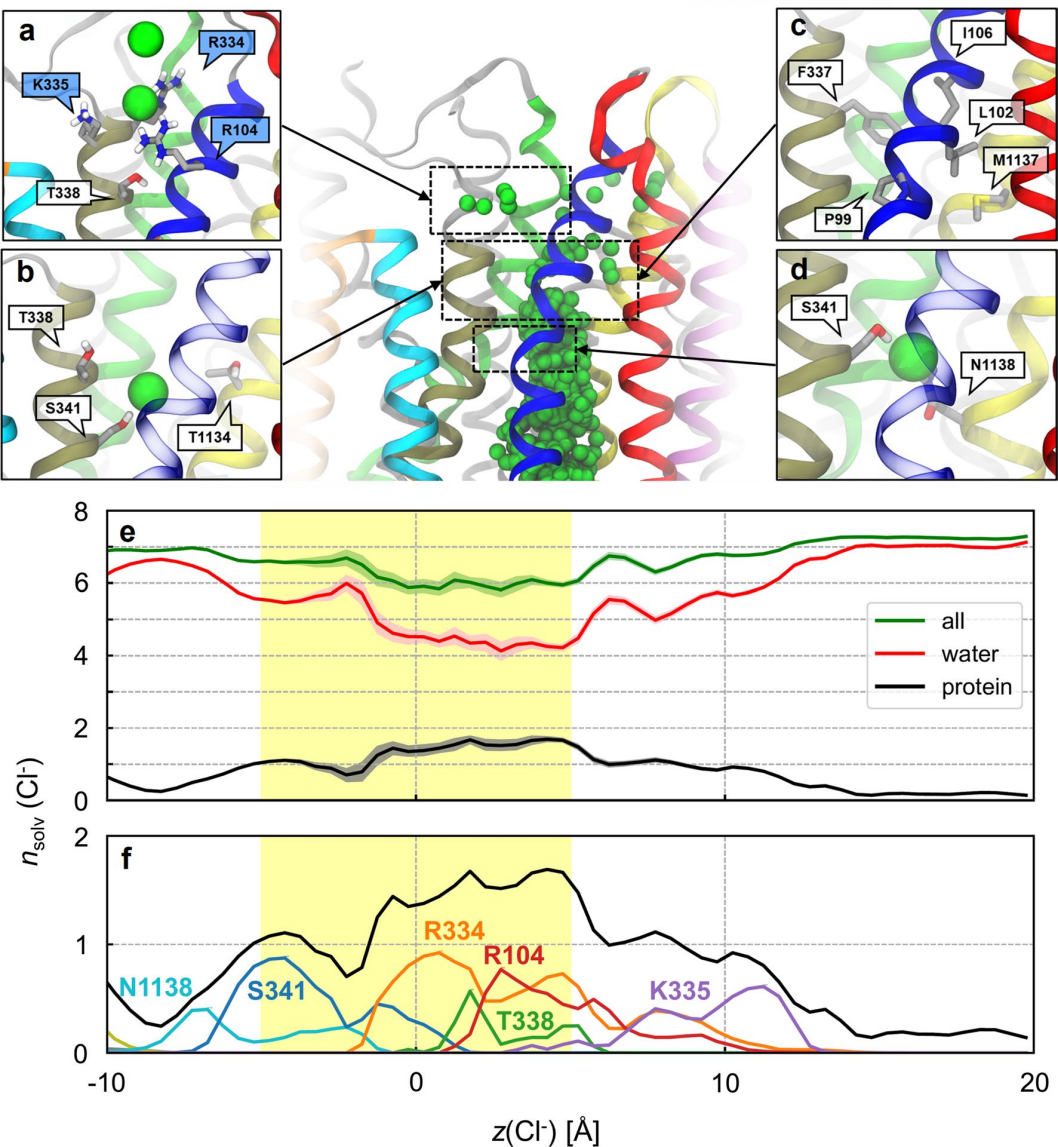
Chloride translocation pathway through the bottleneck region. Top centre: overview of the Cl^−^ permeation pathway with overlaid ions (green spheres) from simulations in the presence of voltage with TM helices 1 (blue), 2 (red), 3 (cyan), 4 (orange), 6 (tan), 8 (lime), 11 (purple), and 12 (yellow). **a**–**d** Close-up views showing the residues interacting with permeating Cl^−^ ions. The labels of positively charged residues are shaded in indigo. **e** Coordination of Cl^−^ ions inside the channel cavity from simulations with voltage. The average numbers of protein residues (black) and water molecules (red) in the first solvation shell of Cl^−^ ions (*n*_solv_) along the intracellular pathway are shown together with their sum (green). Shading represents 99% bootstrapped confidence intervals. Chloride ions are less hydrated in the bottleneck region (yellow shading). **f** Significant amino-acid contributions (various colours) to the protein solvation number *n*_solv_ (black)

The translocation of Cl^−^ through the bottleneck region begins at *z* = − 5 Å. At this location, Cl^−^ ions bind S341 and N1138 from TM6 and TM12, respectively (Fig. 5d). These residues precede the bottleneck region and can be reached by intracellular Cl^−^ ions without leading to translocation, even in the presence of a strong transmembrane voltage (Figs. 4, S6). Beyond S341, translocating Cl^−^ ions traverse the bottleneck region lined by a number of hydrophobic residues including L102, I106, F337, and M1137 (Fig. 5c). Halfway through the bottleneck (*z* = 0 Å), T338 and T1134 mark the narrowest region of the pore and occasionally coordinate permeating Cl^−^ ions (Fig. 5b). The extracellular end of the bottleneck is lined with several positively charged residues, including R104, R117, R334, and K335, which could facilitate the passage of permeating anions through the bottleneck (Fig. 5a). These cationic residues contribute to the partial dehydration of Cl^−^ by displacing on average about 1.2 ± 0.6 water molecules in the first solvation shell of Cl^−^ (Fig. 5e, f). Interestingly, the long sidechain of R334 was even observed to dip into the hydrophobic bottleneck and to bind permeating Cl^−^ ions before they reach T338 (Fig. S7), suggesting that R334 may serve as a chaperone for Cl^−^ translocation.

**Fig. 6:**
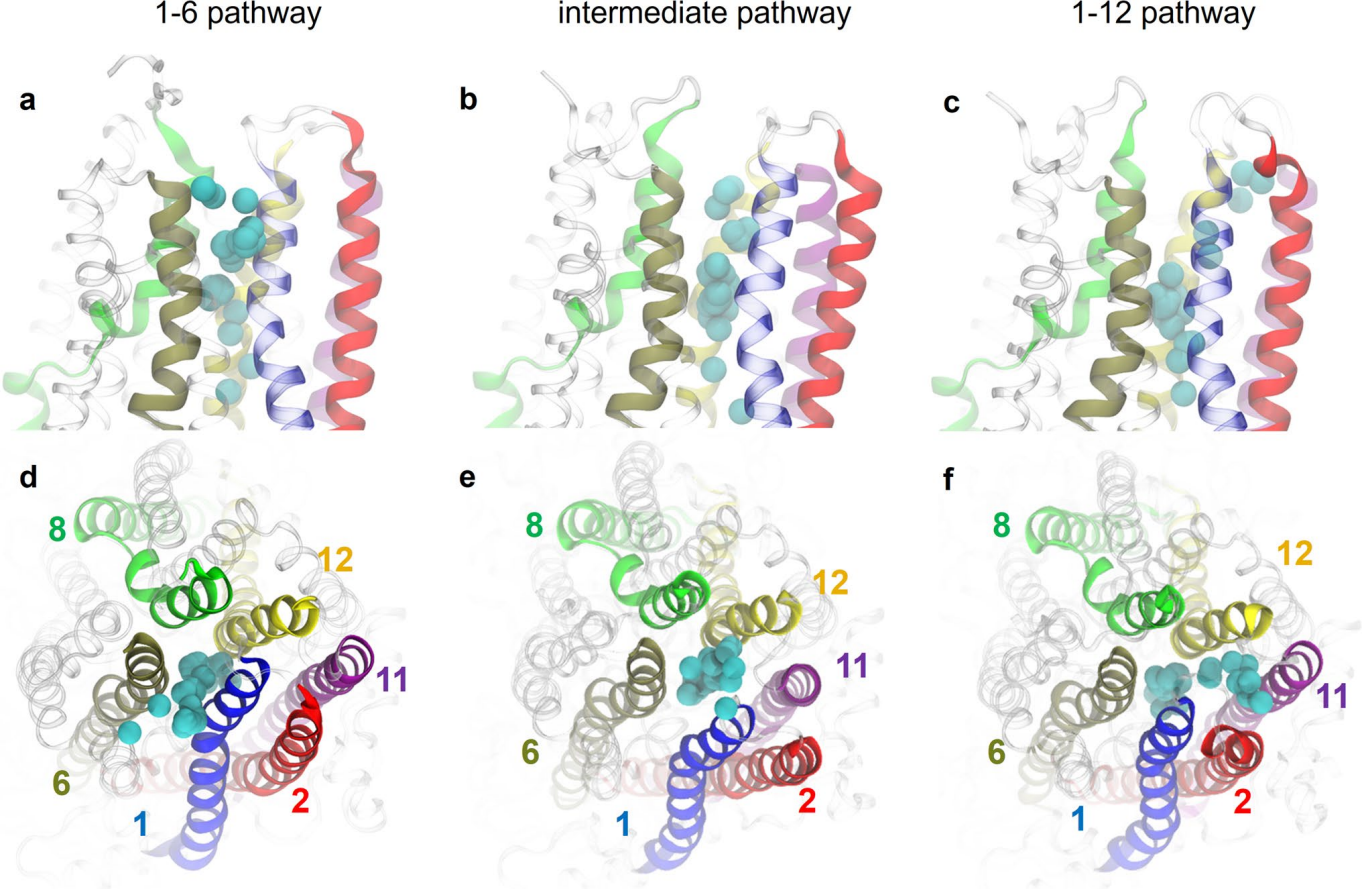
The three different pathways of Cl^−^ permeation through the extracellular bottleneck. (Top row) Side views and (bottom row) top views from extracellular space. Pore-lining helices are colour-coded as indicated. Chloride ions (cyan) overlaid from time steps during translocation. **a**, **d** 1–6 pathway; **b**, **e** intermediate pathway; **c**, **f** 1–12 pathway

**Fig. 7:**
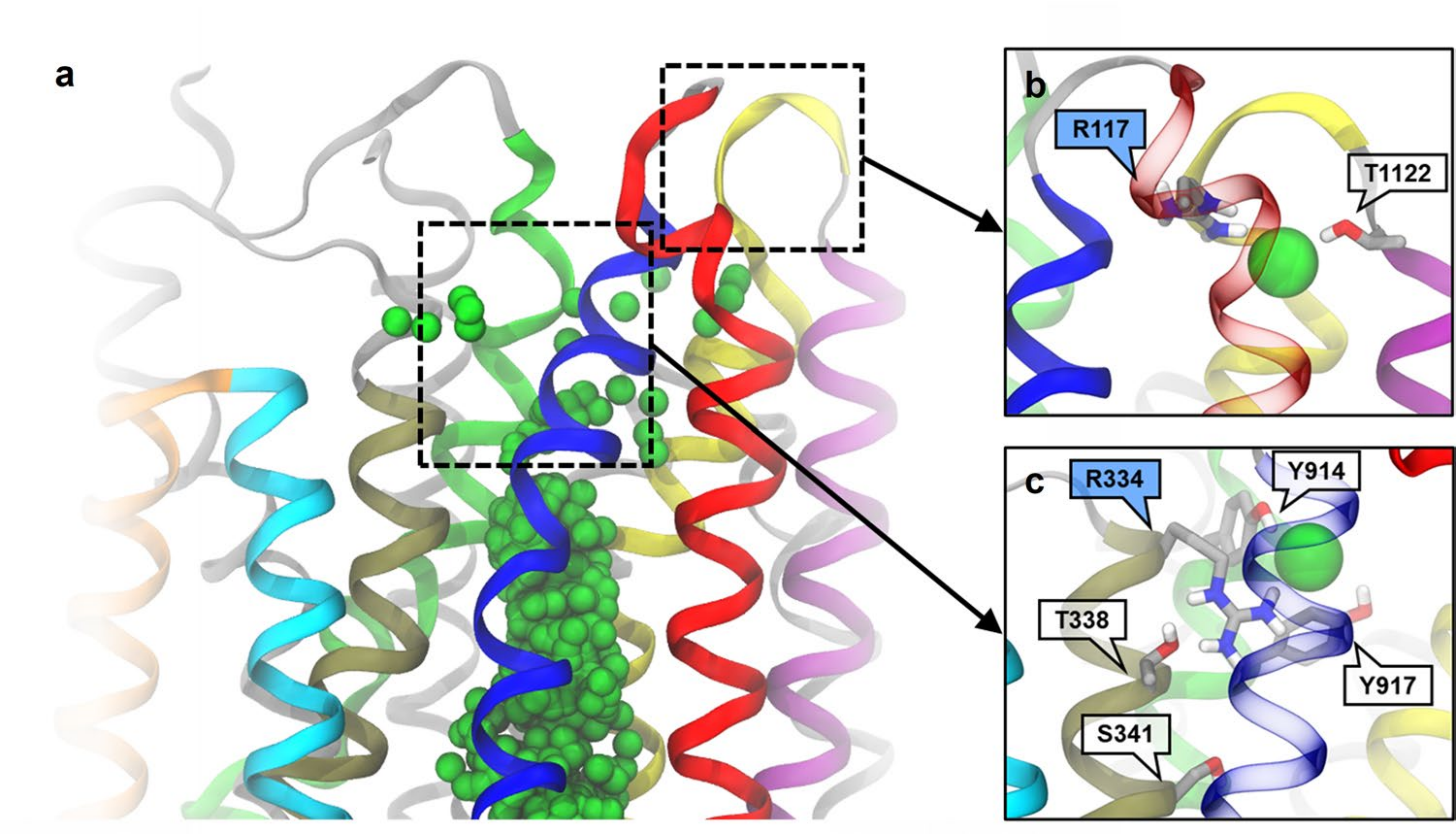
Outer Cl^−^ binding sites at the extracellular mouth. Boxed regions in the overview of the pore **a** are expanded in insets showing Cl^−^ exit binding sites for **b** the 1–12 pathway and **c** the intermediate pathway. TM helices 1 (blue), 2 (red), 3 (cyan), 4 (orange), 6 (tan), 8 (lime), 11 (purple), and 12 (yellow) are highlighted. Residue sidechains interacting with Cl^−^ ions are shown

The Cl^−^ permeation pathway appears to diverge into different routes in the bottleneck region. The point of divergence is located shortly beyond S341. In the first route, Cl^−^ exits laterally between TM1 and TM6; this route will henceforth be referred to as the “1–6 pathway” (Fig. 6a, d). The 1–6 pathway is bounded by TM helices 1, 6, 8, and 12. In an alternative route, Cl^−^ exits between TM helices 1 and 12, which defines the “1–12 pathway” bounded by TM helices 1, 2, 6, 8, 11, and 12 (Fig. 6c, f). The third, “intermediate” permeation route, which is intermediate between the other two, is lined by the same six TM helices in approximate hexagonal arrangement (Fig. 6b, e). The three different permeation routes also differ in which sets of extracellular residues contact translocating Cl^−^ ions: R334, K335, and R104 in the 1–6 pathway (Fig. 5a); R334, Y914, and Y917 in the intermediate pathway (Fig. 7c); R117 and polar groups of extracellular loops in the 1–12 pathway (Fig. 7b).

### Role of cationic sidechains in Cl^−^ permeation

Because of the importance of cationic coordination to Cl^−^ solvation and dynamics, we examined the effect of the applied electric field on the orientation of charged protein residues. To this end, we computed the axial distributions of the centre of charge of each Lys and Arg side chain in the TM region successively with and without applied voltage (Fig. S8). Many of these side chains sample multiple states. Given the direction of the hyperpolarizing electric field, one might expect a systematic negative shift of cationic side chains along the oriented (*z*) axis of the channel. However, the shifts were at most minor and occurred in both directions: while some residues underwent small negative *z* shifts on average (e.g. K95, R242, R1102), others shifted in the positive direction (e.g. R134, R1048, R1066, R1128). These results indicate that the electric field is not strong enough to bias the orientation of cationic residues systematically. Most notably, the field induced a small extra peak at smaller *z* values corresponding to the dipped or “dunked” state of R334.

The emergence of this dunked state shows that voltage does affect the conformational equilibrium of the R334 side chain—if only indirectly, by promoting the wetted state of the gate and creating enough space for the pore bottleneck to accommodate its guanidinium group. In turn, this finding raises the question of whether this new state is favoured by Cl^−^ proximity as well as applied voltage. To address this question, we computed the joint distribution of the axial position of the centre of charge of the guanidinium group of R334 and that of the nearest Cl^−^ ion found “below” (i.e. further into the pore) that cationic group (Fig. S9). Results show that Cl^−^ and R334 can sample the bottleneck region as an ion pair, although the dunked state of R334 was also observed without Cl^−^ in its immediate proximity. In other words, dunking of R334 sidechain does not require approaching Cl^−^ from inside of the pore. Together, these results support a special role for R334 in the catalysis of Cl^−^ permeation.

### Structural features of the open pore

**Fig. 8:**
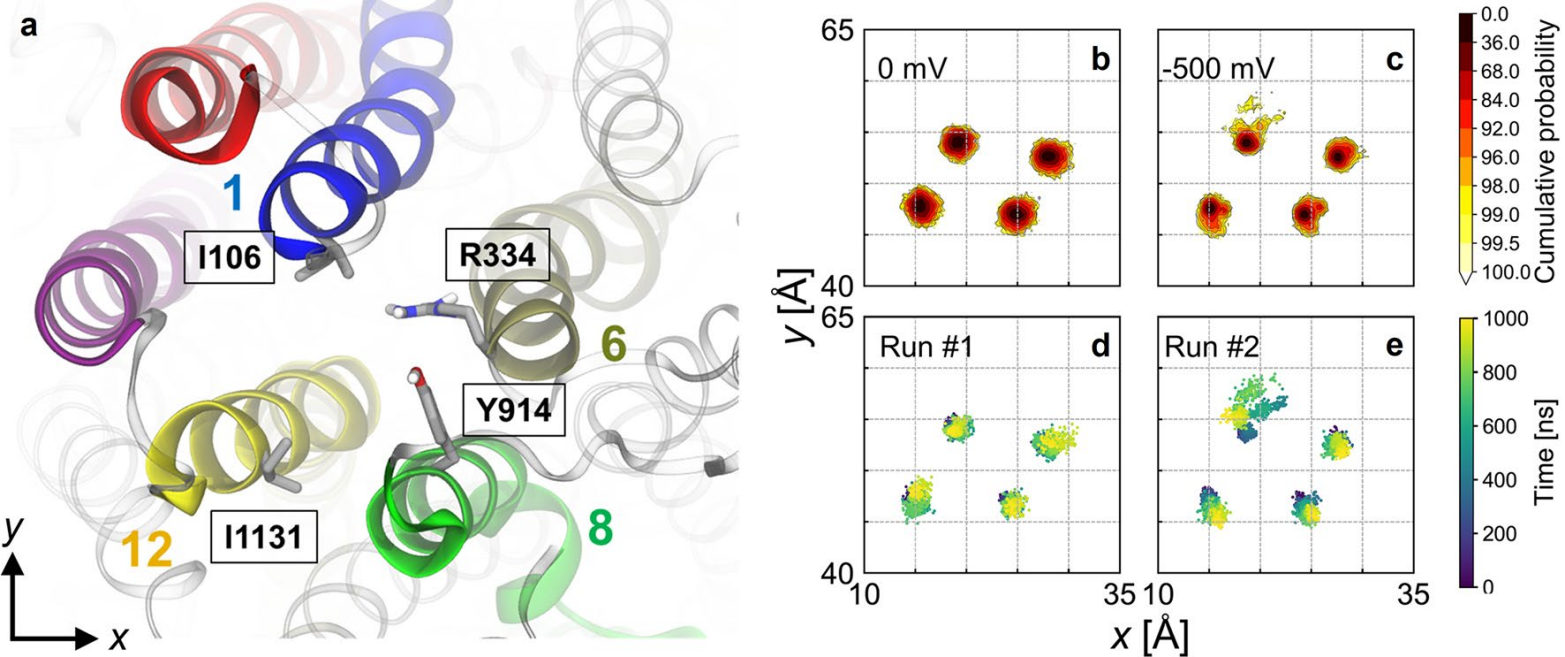
Structural fluctuations of TM helices lining the extracellular bottleneck region. **a** Top view of the pore from the extracellular side highlighting amino acid residues of the pore-lining helices used to track TM helix movements. **b**, **c** Distributions of *xy*-positions of the extracellular ends of TM helices 1, 6, 8, and 12 in the absence and presence of transmembrane voltage. **d**, **e** Time evolution of the lateral positions TM helices 1, 6, 8, and 12 in conductive simulation runs #1 and 2 (− 500 mV). In run #1, a brief departure of TM6 away from the other helices is seen towards 1000 ns. In run #2, TM1 shows significant fluctuations of up to ~ 5 Å from its average location

**Fig. 9:**
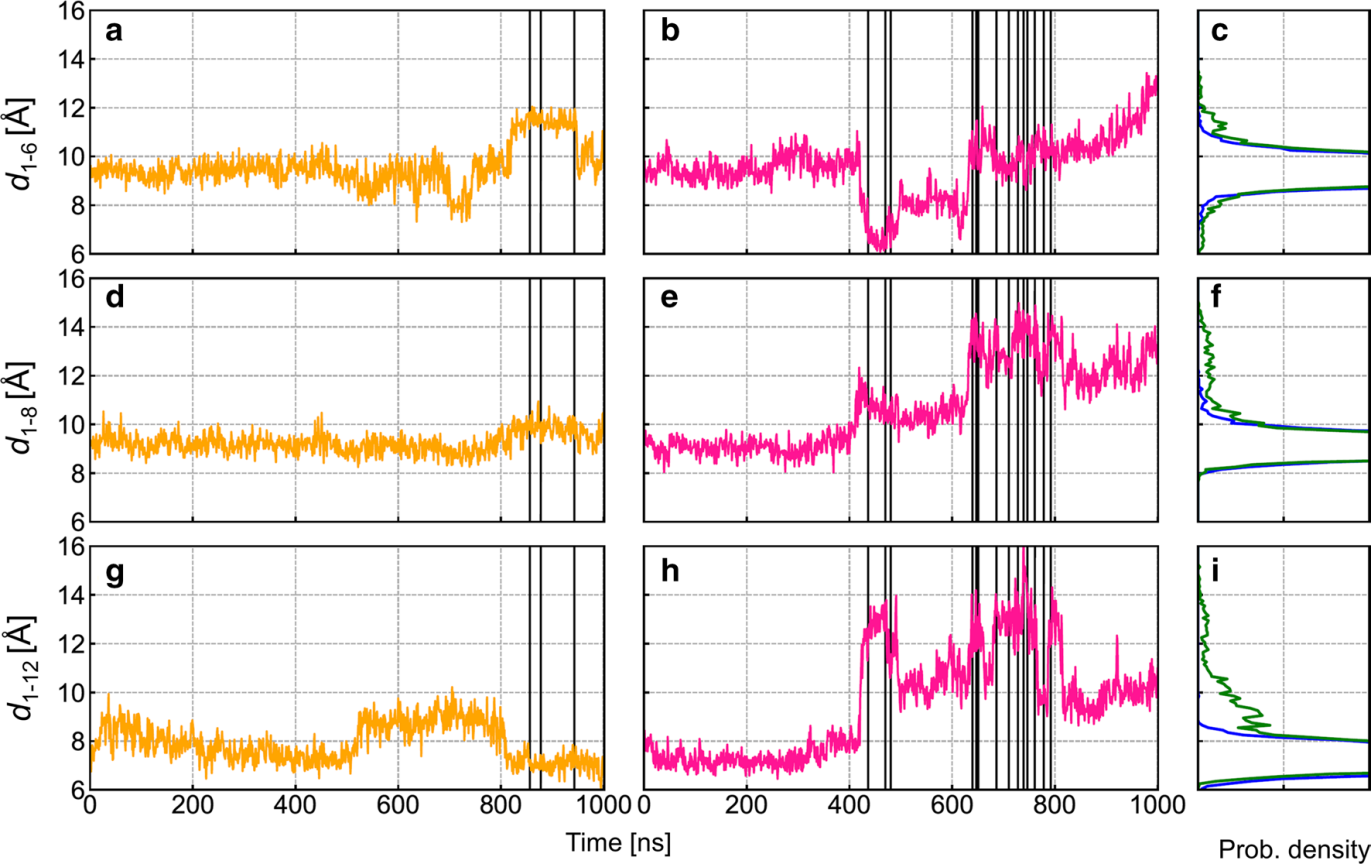
Time evolution of interhelical distances between TM 1 and other pore-lining helices at the extracellular end. Interhelical distances are computed as the distances between Cα atom pairs of the following residues: *d*_1–6_: I106-R334 (top row); *d*_1–8_: I106-Y914 (middle row); and *d*_1–12_: I106-I1131 (bottom row). **a**, **d**, **g** Time series of interhelical distances for run #1 (orange traces); **b**, **e**, **h** same for run #2 (pink traces). Chloride translocation events (see Fig. 4) are indicated as vertical lines. **c**, **f**, **i** Distributions of interhelical distances in the absence (blue) and in the presence (green) of an electric field

In the simulations in which Cl^−^ permeation events occurred, changes in the distances separating pore lining helices in the bottleneck region were correlated with transitions from non-conductive to conductive states (Fig. 8). In run #1, ion permeation may be facilitated by TM6 moving away from other pore lining helices (Fig. 8d). In particular, the distance *d*_1–6_ increased from ~ 9 to ~ 12 Å before the permeation burst starting at 800 ns (Fig. 9a), creating a pore large enough for ion exit through the 1–6 pathway (Table S1).

In run #2, ion permeation was likely facilitated by TM1 moving away from the other pore-lining helices (Fig. 8e). The distances *d*_1–8_ and *d*_1–12_ increased at ~ 400 ns (Fig. 9e, h) even as *d*_1–6_ decreased before reverting to larger values during the second ion burst period (Fig. 9b). The decreased TM1-TM6 separation may explain that all three permeant Cl^−^ ions took the 1–12 pathway during the first ion burst (Table S1), during which *d*_1–12_ peaked (Fig. S10). The second ion burst featured a large *d*_1–12_ and a further increase in *d*_1–8_ (Fig. 9e, h). During this period, permeant Cl^−^ ions followed mostly the intermediate and 1–6 pathways (Table S1). After the second ion burst, *d*_1–6_ increased further while *d*_1–12_ decreased (Figs. 9b, h, S10), suggesting that the channel may be undergoing a transition towards a state in which the 1–6 pathway is the exclusive route, similar to that observed in run #1.

### Hydration of the bottleneck region

**Fig. 10:**
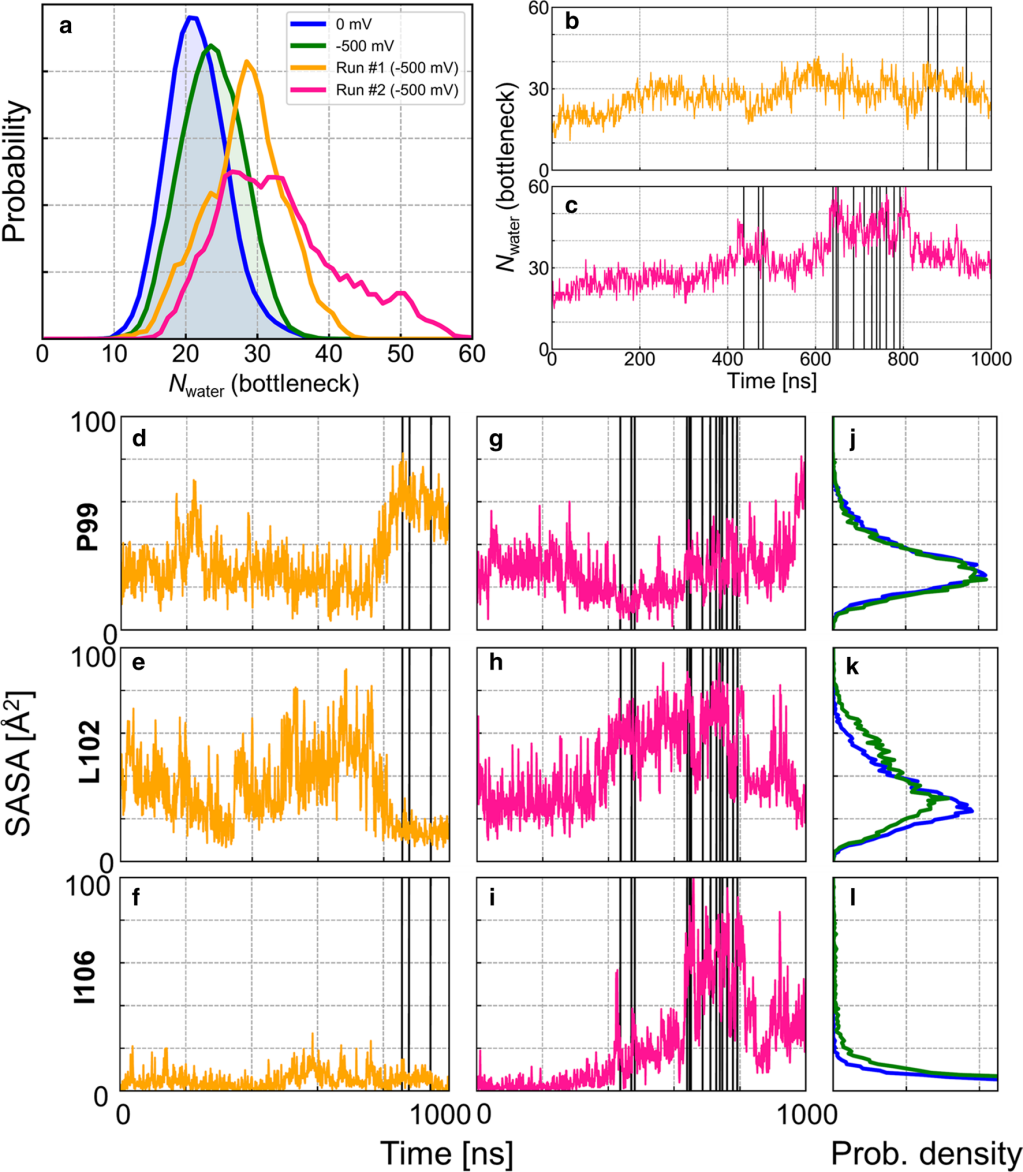
Analysis of hydration of the extracellular bottleneck region. **a** Probability distribution of the number of water molecules in the bottleneck region, *N*_water_(bottleneck), from non-conductive simulations in the presence (green) and absence (blue) of TM voltage. Results computed from conductive simulation runs #1 (orange) and #2 (pink) are shown separately. **b**, **c** Time evolution of *N*_water_(bottleneck) for simulation runs #1 and 2. **d**–**i** Time series of solvent accessible surface area (SASA) of residues P99, L102, and I106 found in the narrowest region of the bottleneck for simulation runs #1 (**d**–**f**) and 2 (**g**–**i**). The time series suggest temporal correlation with ion permeation. **j**–**l** Distributions of SASA of these residues in the absence (blue) and in the presence (green) of TM voltage are shown in the rightmost column. Ion translocation events (see Fig. 4) are indicated as vertical lines

The observation of partially hydrated Cl^−^ ions permeating through the CFTR channel suggests that a hydrated bottleneck region is a prerequisite for ion conduction. Overall, the average number of water molecules in the bottleneck increased slightly when the electric field was present (Fig. 10a), a phenomenon seen in hydrophobic gates and known as “electric field-induced wetting” [42]. In particular, the overall degree of hydration in run #1 and #2 was greater than that of all other simulations conducted with an electric field. All ion permeation events in simulation run #1 occurred when hydration was relatively high (bottleneck *N*_water_ ~ 30) after *d*_1–12_ increased to permit water permeation (Figs. 4, 9g, 10b; water permeation results not shown). Likewise, the time dependence of hydration in run #2 was strongly correlated with ion permeation events. The first and second permeation bursts occurred following major increases in hydration at *t* ~ 400 ns and *t* ~ 600 ns, respectively (Figs. 4, 10c). Permeation occurred during periods of wetting of the bottleneck region, which provided a hydrated pathway for ion translocation.

**Fig. 11:**
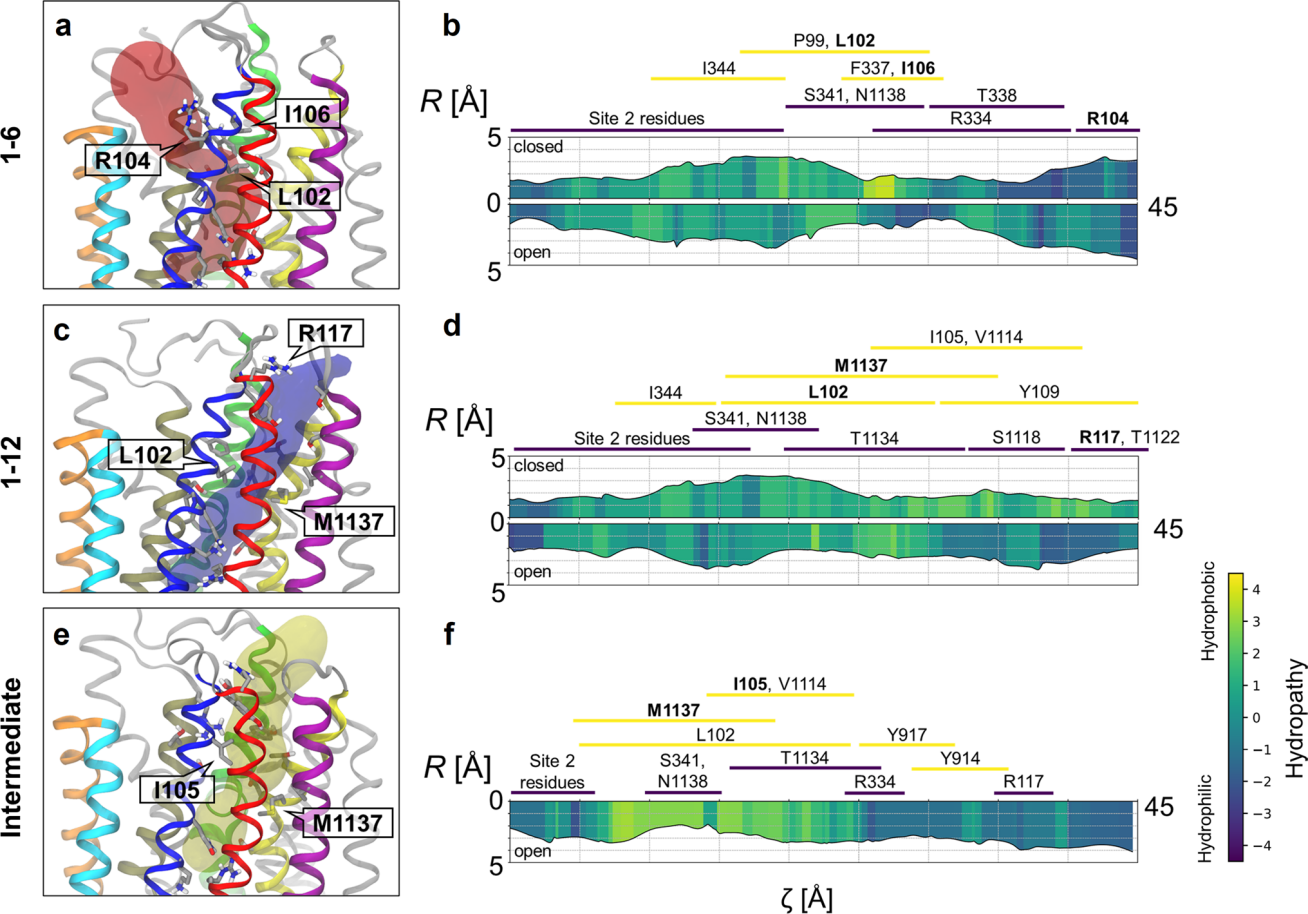
Structural and physico-chemical analysis of diverging permeation pathways through the extracellular bottleneck region. **a** 1–6, **c** 1–12, and **e** intermediate pathways are visualized as translucent closed surfaces. Estimates of width and hydrophobicity along the 1–6 (**b**), 1–12 (**d**), and intermediate pathways (**f**); *R*, local radius and *ζ*, curvilinear distance along the pathway. The comparison of closed and open states shows reduced hydrophobicity and/or increased width of the permeation pathways. Note that the intermediate pathway in the “closed” state could not be detected and analysed. The approximate ranges of pathway-lining residues are indicated for each of the three pathways

The coincidence of pore wetting and ion permeation events is reminiscent of hydrophobic gates in a large variety of other ion channels [43, 44]. To locate a putative hydrophobic gate in CFTR, we analysed hydrophobicity along the permeation pathways. A short but highly hydrophobic “gasket” consisting of residues L102, I106, and F337 constitutes the most hydrophobic and narrowest region within the 1–6 pathway (Fig. 11a, b). The hydrophobicity of this region decreases drastically when sustained hydration and permeation occur due at least in part to the penetration of the side chain of R334 (Fig. S7). Similarly, residues L102, I105, and M1137 constitute a highly hydrophobic region within the intermediate pathway (Fig. 11e–f). In contrast, the 1–12 pathway does not have a distinct, sharply hydrophobic region; instead, the entire pathway is somewhat hydrophobic (Fig. 11c, d). During permeation, the entire pathway appears widened, with the narrowest region widening by about 1–2 Å in diameter.

Drastic changes in hydration around the gasket region occurred during transitions between non-conducting and conducting states (Fig. 10d–i). In run #1, P99 and L102 became substantially more and less hydrated, respectively, prior to the ion permeation burst (Fig. 10d, e). Since L102 was much more water accessible when water permeation occurred through the 1–12 pathway (Fig. 10e; 500 < *t* < 800 ns), the decrease in water accessibility of L102 might coincide with the closure of the 1–12 pathway and the opening of the 1–6 pathway. These observations suggest that differential extent of wetting of these residues is correlated with the availability of different permeation pathways. Accordingly, L102 is more water exposed than P99 when Cl^−^ permeation occurs through the 1–12 and intermediate pathways in run #2 (Fig. 10g, h). Furthermore, I106 is more hydrated in run #2 than in run #1, especially during the second ion permeation burst (Fig. 10i), which may be a hallmark of a wide-open gate supporting all permeation routes. In summary, wetting of hydrophobic residues within the bottleneck region precedes permeation events regardless of permeation routes; however, which residues undergo wetting depends on the permeation pathway being activated.

## Discussion

### Effect of applied voltage

The primary purpose of this study was to obtain a model of the open state of the CFTR channel from the cryo-EM structure of dimerized, ATP-bound human CFTR. This objective was achieved by embedding the channel in a phospholipid bilayer and by imposing a membrane voltage, which helped the closed conformation of the channel relax to an open, ion-conducting state. Applying an electric field to create a transmembrane voltage is a methodology often used in MD simulations of ion channels to speed up events occurring over longer biological timescales. Here, the relatively large magnitude of the applied voltage (0.5 V) was not meant to mimic physiological conditions, but to facilitate or accelerate the conformational relaxation of the pore to the open state, an approach successfully used in a recent study of HCN channels with applied voltages of − 550 to − 750 mV [38] or in an older study of a K^+^ channel [37]. Although voltages of such magnitude are not physiological, they are also routinely used in MD simulation studies of ion channels to speed up ion translocation events. Examples include studies of voltage-gated Na^+^ channel NavMs [45] and of K^+^ channel MthK [46].

Consistent with the stochastic nature of closed-to-open conformational relaxation observed in other MD studies of ion channels [33], the channel only reached a functionally conductive state in two out of ten microsecond-long simulation repeats. In these two simulations, ion translocation events were frequent and likely accelerated by the magnitude of the electric field. Importantly, the structure and fluctuations of the CFTR channel were not otherwise systematically affected by the high applied voltage.

### Comparison with previous structural models of the open state

Numerous in silico studies involving molecular simulations of homology models of CFTR have aimed to provide structural models of the channel in its open conducting state (reviewed in [47]). However, a major limitation of these models is the use of homologous template ABC transporters sharing low sequence identity with CFTR [48–51]. In addition, an unexpected feature revealed by the near-atomic resolution cryo-EM structures is that TM8 is partially unwound in the middle of the membrane and split into two helical segments [19]. As a result, the extracellular segment of TM8 participates in lining the pore, in stark contrast to typical, symmetric ABC transporters in which TM7, not TM8, forms part of the translocation pathway. In support of this finding, Negoda and coworkers, using substituted cysteine accessibility mutagenesis and cysteine crosslinking, found that TM8 contains residues lining the narrow region of the pore in close proximity to TM1 and TM6 in an open CFTR channel [52]. This feature of TM8 is consistent with the near-atomic resolution experimental structures but is not captured by existing homology models.

Our results confirm that the broken TM8 is a stable structural feature of the pore. The unexpected helix break led to speculation that it plays a role in the gating of CFTR [19, 35]. It was proposed that movement of the top segment of the broken helix is the missing step for the cryo-EM structure with ATP and dimerized NBDs to reach the open state [21]. Notably, unlike other structures of ATP-bound CFTR with dimerized NBDs, a lower-resolution cryo-EM structure of chicken CFTR in an ATP-bound form determined to have a high channel opening probability does not contain a broken TM8 [53]. In the present study, the conformation of TM8 in the near-atomic resolution cryo-EM structures was conserved over microseconds of simulation time. This observation is consistent with a previous MD simulation study of zebrafish CFTR, supporting the conformational stability of broken TM8 [35]. In addition, our results also show that, contrary to the earlier proposal [21], pore opening does not require any significant conformational change of TM8 (Fig. S1).

### Comparison with a previous simulation study of ion permeation

A recent MD study by Farkas *et al.* examined the pathway of Cl^−^ permeation in dimerized and ATP-bound zebrafish CFTR whose structure, like that of human CFTR, appears to be closed [36]. In conventional, unbiased simulations, structural fluctuations of the pore led to putative open conformations in which the bottleneck was wide enough to accommodate a Cl^−^ ion. However, that assessment was based on the diameter of the largest cavity connecting cytoplasmic and periplasmic sides of the pore, not on pore hydration or ion translocation events. Furthermore, the putative open state was only reached transiently, with sub-nanosecond lifetimes, and not described in detail. In contrast, the functionally open state of the channel obtained in the present study supports sustained wetting of the hydrophobic gate and ion permeation events hundreds of nanoseconds.

In order to identify ionic permeation pathways through the gate, Farkas* et al.* used metadynamics, a simulation methodology involving biasing potentials to force uniform sampling of Cl^−^ ions across the pore bottleneck [36]. We report spontaneous translocation events induced by a transmembrane voltage rather than by a non-physical biasing potential and we describe the dynamics of ion translocation, information that is not available from metadynamics simulations. Aside from basic differences in methodology and scope, the two simulation studies present notable similarities and differences in ion permeation pathways which are discussed below.

### Chloride binding sites

The location and physico-chemical nature of Cl^−^ binding sites in CFTR have been somewhat contentious [54, 55]. Experimental studies conducted with negatively charged channel blockers point to the presence of anion binding sites inside the cavity [56, 57]. From our simulations, we identified two major binding sites that are consistent with the experimental findings: site 1, near the cytoplasmic portal, consisting of residues K190, R248, and R303; and site 2, deep inside the inner vestibule of the pore, consisting of K95, Q98 and R134 (Fig. 3a). Many of these residues, especially K95 and R134, were shown to form frequent close contacts with Cl^−^ ions in the MD simulation study of zebrafish CFTR by Farkas and coworkers [36]. Several of the residues that contribute to these two binding sites also have previously been shown to interact with channel-blocking anions, which have been suggested either to occupy a superficial site close to the cytoplasmic portal [40, 57] or to enter deep into the cavity and interact with the positive charge of K95 [56]. Furthermore, the reduced conductance associated with mutations that remove positive charges in these binding sites suggests that Cl^−^ binding to these sites is an important feature of its permeation mechanism [41, 58, 59]. These two inner binding sites are located in regions where Cl^−^ occupancy is likely to be important–namely, at the cytoplasmic entrance of the pore (site 1) and near the entrance of the bottleneck (site 2). Indeed, Cl^−^ ions are most likely to occupy site 1 regardless of electric field, while the presence of an electric field increases the affinity of the middle of the pore for anions, as seen by the increase in Cl^−^ occupancy at site 2 (Fig. 2d, e).

Even though simultaneous binding to multiple sidechains of site 2 does occur occasionally, usually only one residue binds Cl^−^ at a time, suggesting that Cl^−^-binding residues are loosely organized at the binding site. Accordingly, it was found by mutagenesis that the positive charge of K95 can be moved to a nearby location, such as I344, V345, or M348, without abrogating channel function [60, 61]. Similarly, the positive charges on K190 and R303 can be moved to nearby locations, such as N186 and L197, with minimal reduction in Cl^−^ conductance [62].

Previous MD simulations of zebrafish CFTR by Farkas and coworkers [36] suggested two lateral cytoplasmic portals through which Cl^−^ ions can potentially enter: one formed by TM4 and TM6 and lined by cationic residues corresponding to K370, R248, and K190 in human CFTR; the other formed by TM10 and TM12 and lined by cationic groups corresponding to K1041 and R1048. While the TM4-TM6 portal was suggested to be the main entrance, it was not reported whether Cl^−^ ions entered through both portals. In our simulations of human CFTR, Cl^−^ ions entered the inner vestibule exclusively through the TM4-TM6 portal (Fig. S4) although K1041 and R1048, residues thought to potentially attract Cl^−^ ions from the intracellular space, constitute an off-pathway binding site next to site 1. In addition, our own previously unpublished simulations of zebrafish CFTR show that Cl^−^ indeed entered through both of the portals identified by Farkas *et al.*, suggesting that this discrepancy might be due to subtle differences between the zebrafish and human CFTR structures (see Supplementary Methods and Fig. S4).

### Ion permeation through the bottleneck region

Consistent with functional and SCAM studies, in the ion-conducting conformations of CFTR, the ionic pathway through the bottleneck is lined primarily by TM helices 1, 6, 8, and 12 (Fig. 1c) [52, 54, 63]. TM helices 2 and 11 also contribute to the permeation pathway, albeit to a lesser extent (Figs. 1c, 6). While there has been some evidence suggesting that TM11 lines the narrow region of the permeation pathway, evidence supporting the same role for TM2 is lacking [54]. Our simulations show two key residues by which TM2 could functionally contribute to the Cl^−^ permeation pathway: R134, which contributes to binding site 2; and R117, which binds Cl^−^ ions permeating through the 1–12 pathway. However, neither of these residues is located within the narrow bottleneck region. Furthermore, neither TM2 nor TM11 appear to make any contribution to the 1–6 pathway.

The simulations show that Cl^−^ entering the bottleneck region from the intracellular side first contact S341 (Fig. 5d, f). The hydroxyl group of S341 participates in Cl^−^ solvation, which might be important given that the S341A mutant is associated with a large decrease in Cl^−^ conductance [64]. Moreover, mutation S341K also causes greatly reduced conductance, highlighting the importance of this side chain to the permeation process [61]. Beyond S341, the permeation pathway is lined by hydrophobic sidechains including L102, I106, F337, and M1137 (Fig. 5c). Functional and SCAM data suggest that L102 and F337 contribute to the narrowest part of the open pore [65, 66]. Mutations of T338 show strongly size-dependent effects on conductance, consistent with the observation that this residue is located in the narrowest part of the bottleneck [67, 68]. At the extracellular end of the bottleneck, Cl^−^ ions interact with R104, R117, R334, and K335 (Figs. 5a, 7b, c). The positive charges at R104, R334, and K335 have each been shown to interact with extracellular anions, and charge-neutralizing mutations at these residues reduce Cl^−^ conductance [63]. Finally, Cl^−^ ions exiting via the intermediate pathway interact with Y914 and Y917, mutations of which were shown to affect both Cl^−^ permeation and channel gating [52].

Longstanding functional evidence supports an important role for R334 in Cl^−^ permeation [69, 70]. Accordingly, our simulations show that R334 binds permeating Cl^−^ ions (Figs. 5f, 7c). Moreover, its sidechain undergoes conformational isomerization between an out-facing state and a dunked state in which it assists Cl^−^ translocation through the bottleneck (Fig. S7). The latter finding is consistent with cysteine accessibility data showing loss of extracellular access to this residue when the channel opens [71, 72]. As such, R334 dunking provides an explanation for the apparent paradox of the reduced accessibility of this pore-lining sidechain in the open state.

Structural fluctuations leading to the open conformation of the channel involve relative movements of TM1 and TM6 (Fig. 8). Cysteine cross-linking studies suggested that the extracellular ends of TM1 and TM6 separate from each other when the channel opens [73], which is consistent with the 1–6 permeation pathway observed in this study. TM1 movements best explain gate wetting and ion translocation. These movements may be facilitated by a helical defect at residue P99. Consistent with this hypothesis, mutations that favour helicity, such as P99A and P99L, are both loss-of-function mutations [74]. TM6 movements may also facilitate ion translocation, as seen in run #1, albeit to a lesser degree. In contrast, we did not observe noticeable movements of TM8 and TM12 as proposed by Zhang and coworkers [21]*.*

In their MD simulation study of zebrafish CFTR, Farkas* et al.* [36] observed that a phospholipid tail in the vicinity of TM8 intruded into the bottleneck region, where it may block the Cl^−^ permeation pathway. In our simulations, we did not observe any direct contribution of lipids to the ion pathway, either in the bottleneck region or elsewhere in the pore. In addition, we also did not find systematic evidence for a defect in the lipid/water interface in the vicinity of the E873-R933 ion pair, two residues located on TM7 and 8, respectively, as reported by Corradi* et al.* in their study of zCFTR [35]. More specifically, the defect only occurred when the two residues did not form a salt bridge when the protein was embedded in a bilayer.

### Putative explanations of lyotropic permeability selectivity

Like most anion channels, CFTR shows lyotropic permeability selectivity, meaning that lyotropic anions with a relatively low free energy of hydration have higher permeability than kosmotropic anions, which retain water in their hydration shell more strongly [75, 76]. One implication of lyotropic permeability selectivity is that partial dehydration is an important aspect of the anion permeation process. Consistent with this hypothesis, Cl^−^ ions lose water molecules from their first solvation shells as they pass through the bottleneck region (Fig. 5e). This finding suggests that the bottleneck might form the lyotropic “selectivity filter,” where the relative permeability of different anions is predominantly determined. Amongst all the pore-lining residues studied, only mutagenesis of F337 and, to a lesser extent, L102, have been shown to disrupt the normal lyotropic selectivity pattern [66, 76]. These non-polar residues form a narrow, hydrophobic region of the open pore where Cl^−^ ions are partially dehydrated (Fig. 5e, f). It is conceivable that partial dehydration might favour the entry (and therefore permeation) of more lyotropic anions, thus explaining the lyotropic permeability selectivity pattern.

### Diverging ion translocation pathways in the bottleneck region

Our simulations reveal multiple Cl^−^ exit pathways to the extracellular space (Fig. 6). In support of both 1–6 and 1–12 pathways, charge-neutralizing mutations at R104, R334, K335, and R117 on the extracellular side lead to reduced channel conductance [70, 77]. In addition to these two permeation pathways, a less distinguishable, intermediate pathway (Figs. 6b, e, 11e) involving direct Cl^−^ coordination by tyrosine sidechains Y914 and Y917 was observed when the gate was most widely open (Fig. 7c). In support of the intermediate pathway, a number of mutations of these tyrosine residues lead to reduced channel conductance [52].

Given the small number of ion permeation events in the simulations, we cannot assess the relative stability and functional importance of each pathway. The existence of these different pathways may not correspond to observable changes in the functional properties unless specific blockage of individual putative pathways can be achieved. Mutagenesis of some residues in the outer mouth of the pore, including R117, had relatively minor effects on conductance, possibly because these residues contribute to the pathway taken by only a subset of all permeating Cl^−^ ions [77, 78]. Furthermore, rapid fluctuations in the conformation and relative arrangement of pore-lining helices governed the transitions between the different pathways over a 100-ns timescale (Table S1: simulation run #2: 600 < *t* < 800 ns). One implication of TM-helix movements is that the extracellular part of this pore is structurally dynamic, which might explain why we did not identify a clearly defined “outer vestibule” region as suggested in the classical description of the architecture of CFTR pore [63].

### A hydrophobic gate with unusual features

In a previous computational study, a heuristic method was used to estimate the hydration state of the pore and predicted that the cryo-EM-derived conformations of both zebrafish and human CFTR contain a dewetted hydrophobic gate and are therefore closed [32]. The estimate was based on static structures (the PDB structure) and did not incorporate thermal fluctuations or explicit water molecules. Our results confirm their prediction and provide further evidence that the extracellular bottleneck region of the pore functions as a hydrophobic gate. Hydration of the hydrophobic bottleneck was induced by the presence of the electric field (Fig. 10a, j–l). Electric-field-induced permeation of water and ions was observed in previous simulation studies of hydrophobic nanopores [79]; more recently, wetting of hydrophobic gates was demonstrated in simulations of biological ion channels in the presence of a strong electric field [39, 42, 80]. It has been suggested that the presence of an electric field can alter the liquid–vapour equilibrium of water inside hydrophobic gates, resulting in an increased probability of wetting [39]. This effect might have contributed to water and ion permeation in our simulations. However, the wetting transition observed in the simulations can also be explained in terms of structural changes. Increasing both the polarity and the diameter of the gate are common features of hydrophobic gating [81]. In our simulations, both of these events occurred due to movements of helices and changes in sidechain conformations, both of which may be induced by the electric field.

Despite similarities to other hydrophobic gates, the gate of CFTR also presents some peculiarities. First, it is not distinctively shaped due to the presence of multiple permeation pathways. While the 1–6 pathway resembles a typical ion channel pore with a short hydrophobic segment, the 1–12 pathway does not seem to have a distinct gating region, yet it is overall lacking in polar residues and, as expected, it undergoes dilation prior to ion permeation. Second, partial dehydration of the permeant ion occurs as it passes through the hydrophobic bottleneck. The fact that sidechains such as R334 can participate in the solvation of permeant ions complicates its categorization as a hydrophobic gate. Curiously, size-reduction mutation F337A and hydrophobicity-reducing mutation L102T both result in reduced channel conductance [58, 66]. Together with other intriguing effects of mutations in residues of the bottleneck region [55, 63], these are some of the transport properties of Cl^−^ in CFTR that remain to be unravelled.

## Conclusions

This study provides, for the first time, a detailed structural model of the open state of human CFTR consistent with a recent cryo-EM structure of the channel. Repeated microsecond-long simulations performed in the presence of transmembrane voltage led to the spontaneous opening of the channel from the cryo-EM-determined structure. This conformational relaxation consisted of a rearrangement of TM helices 1 and 6, which line the hydrophobic bottleneck in the extracellular region of the pore. Consistent with a hydrophobic gating mechanism, changes in the size, shape, and polarity of the bottleneck resulting from this structural relaxation gave rise to wetting of the gate, which led to spontaneous Cl^−^ translocation events.

Permeating Cl^−^ ions interact with pore-lining residues that are critical for ion conductance, supporting this model of open CFTR. Due to the relatively small number of ion permeation events observed over the course of the two simulations in which the channel reached the open state, our results do not afford a quantitative estimate of ion permeation properties. Longer simulations of the open state over a range of lower, physiological voltages will be required to compute steady-state current–voltage relationships of human CFTR, from which quantitative estimates of Cl^−^ conductance can be obtained and compared to single-channel conductance measurements. These future studies will aim to clarify the relative stability of the diverging permeation pathways observed in the present study and provide further validation of our structural model of the conducting state of CFTR.

## Supplementary Information

Below is the link to the electronic supplementary material.

Supplementary file1 (PDF 5323 KB)

## Data Availability

The MD simulation datasets are available from the corresponding author(s) upon reasonable request.

## Abbreviations

MD: Molecular dynamics
CF: Cystic fibrosis
CFTR: Cystic fibrosis transmembrane conductance regulator
ATP: Adenosine triphosphate
ABC: ATP-binding cassette
TM: Transmembrane (helix if followed by a number)
TMD: Transmembrane domain
NBD: Nucleotide-binding domain
SASA: Solvent-accessible surface area
SCAM: Substituted cysteine accessibility method

## Code availability

Selected analysis scripts, parameter files used to reproduce the MD simulations, and parameter files used in MOLE2 analysis will be provided through Zenodo (DOI: 10.5281/zenodo.7553072).
