# Supplementary material for: Molecular dynamics study of Cl− permeation through cystic fibrosis transmembrane conductance regulator (CFTR)
Source: Cell Mol Life Sci. 2023 Jan 24;80(2):51. doi: 10.1007/s00018-022-04621-7 (PMC9873711; doi:10.1007/s00018-022-04621-7)
Supplement: Supplementary file 1 — Supplementary file1 (PDF 5323 KB) [file 18_2022_4621_MOESM1_ESM.pdf]

Supplementary Information to:

Molecular Dynamics Study of Cl^- Permeation through Cystic Fibrosis Transmembrane Conductance Regulator (CFTR)

Cell and Molecular Life Sciences

Zhi Wei Zeng, Paul Linsdell, Régis Pomès

Supplementary Methods:

1. Simulations of zebrafish CFTR

1.1 Molecular System

The structural model of phosphorylated, ATP-bound zebrafish E1372Q CFTR (PDB: 5W81) was used [1]. Residues missing from the PDB structure include the loops connecting each TMD-NBD pair (406-436, 1182-1202), the R-region (642-843), the extracellular loop between TM7 and TM8 (887-917; also known as the extracellular loop 4), and the segment at the C-terminal end of NBD2 (1459-1485). These missing segments were not modelled in this study, so that our structural model consisted of five peptide chains. All 5 of these chains were acetylated at the N-termini and amidated at the C-termini into primary amides. Mg-ATP moieties at the NBD interface were retained in the simulations. Unlike in the human CFTR PDB structure (6MSM), cholesterol, phospholipids, and the helix at the TMD-NBD interface were absent in the zebrafish CFTR PDB structure.

The PPM server of Orientations of Proteins in Membranes (OPM) database was used to determine the starting position of the lipid bilayer with respect to the CFTR protein [2]. The CFTR protein with Mg-ATP bound was then embedded in a POPC bilayer using InflateGRO2 [3]. The Perl script of InflateGRO2 used was slightly modified to enable the use of a hexagonal simulation box. The hexagonal periodic unit cell configuration was chosen with starting dimensions: $a = b = 11$ nm, $c = 18$ nm, $\alpha = \beta = 90°$, and $\gamma = 120°$. This procedure resulted in a total of 262 POPC molecules added around the protein. GROMACS 2018 was used to add SPC216 water to the system [4]. Water molecules within the lipid bilayer and the transmembrane region of the channel pore were removed

using VMD [5]. GROMACS was used to add 150 mM excess NaCl to the system along with neutralizing the charge of the system with the same ions.

1.2 Simulation setup and protocol

All MD simulations were conducted using GROMACS 2018 [4]. The CHARMM36 forcefield was used for protein, lipids, ions, and ATP, together with the TIP3P water model [6–9]. Simulations were run in the *NpT* ensemble (T = 300 K, p = 1 atm) at 2 fs integration timesteps. Constant temperature was maintained using the Nosé–Hoover thermostat (τ_T = 0.5) [10, 11]; constant pressure was maintained using the Parrinello-Rahman barostat (τ_p = 2.0) [12, 13]. Semi-isotropic pressure coupling was used, with isothermal compressibility set to 4.5×10^{-5} bar^{-1} both in the *xy*-plane and along the *z*-axis. Nonbonded interactions were calculated using Verlet neighbor lists [14, 15]. Lennard-Jones interactions were cut off at 1.2 nm and a force-based switching function with a range of 1.0 nm was used. The particle-mesh Ewald (PME) method was used to compute electrostatic interactions with a real-space cut-off of 1.2 nm [16, 17]. The LINCS algorithm was used to constrain covalent bonds involving H atoms [18].

The simulation system was first subjected to steepest descent energy minimization until maximum force dropped below 1000 kJ/mol/nm. Random velocities were generated at the beginning of the *NpT* equilibration phase, which was conducted in three 10-ns stages, successively with protein heavy atoms, protein backbone atoms, and protein Cα atoms restrained (with force constants of 1000 $kJ/mol/nm^2$ in *x*, *y*, and *z* directions). Following the *NpT*-equilibration phase, the production phase of the run was conducted in *NpT* ensemble at the same temperature and pressure without applied restraints. New random velocities were generated at the beginning of production runs. Ten 1-μs-long simulations were produced for this system.

2. Analysis of durations of ion binding at sites 1 and 2

2.1 Determination of Cl^- ion binding durations

Ions were considered to be in the channel pore if they were within the boundaries of the cylinder constructed based on the coordinates of the Cα atoms of pore-lining helices, as described in the main Methods section. Only time periods during which Cl^- ions resided

continuously in the channel pore (without exiting) for at least 50 ns were considered for the following analysis. Sites 1 and 2 are defined as -40 Å ≤ z ≤ -30 Å and -20 Å ≤ z ≤ -10 Å, respectively. We also defined three transition regions: -42.5 Å ≤ z ≤ -40 Å, -30 Å ≤ z ≤ -20 Å, and -10 Å ≤ z ≤ -5 Å. Whenever an ion departed from a binding site and entered a transition region before returning to the original binding site it came from, the time spent in the transition region was added to the binding event at the site.

2.2 Calculation of time constants of ion binding

Assuming ion unbinding to be a first-order exponential decay process, the probability of an ion that was bound at t=0 to remain bound to the site at time t is $P(t) = e^{-\frac{t}{\tau}}$, where τ is the time constant. It follows that $1 - e^{-\frac{t}{\tau}}$ is the probability of the ion remaining bound for a duration t' such that $0 < t' < t$. Hence,

$$1 - e^{-\frac{t}{\tau}} = \int_0^t p(t')\, dt' \quad (1)$$

where $p(t)$ is the probability density function of the binding duration t. It follows that

$$p(t) = \frac{1}{\tau} e^{-\frac{t}{\tau}} \quad (2)$$

Using the method described in 2.1, the durations of all binding events are collected. The binding durations were summarized in a probability density histogram, which is an estimate of the probability density function $p(t)$ (see Fig. S2). We computed two estimates of average binding duration τ using two different approaches. The first estimate (τ_{avg}) corresponds to the average binding durations obtained from the above analysis. The average duration of binding is an estimate of τ, since the expectation value of binding duration $\langle t \rangle$ is:

$$\int_0^\infty t p(t)\, dt = \tau \quad (3)$$

The second estimate (τ_{fit}) was obtained by exponential fit to the probability density histogram using equation (2). Note that these methods of estimation assume that the probabilities of Cl^- binding at sites 1 and 2 are mutually independent.

References:

1. Zhang Z, Liu F, Chen J (2017) Conformational Changes of CFTR upon Phosphorylation and ATP Binding. Cell 170:483-491.e8. https://doi.org/10.1016/j.cell.2017.06.041
2. Lomize MA, Pogozheva ID, Joo H, et al (2012) OPM database and PPM web server: resources for positioning of proteins in membranes. Nucleic Acids Res 40:D370–D376. https://doi.org/10.1093/nar/gkr703
3. Schmidt TH, Kandt C (2012) LAMBADA and InflateGRO2: Efficient Membrane Alignment and Insertion of Membrane Proteins for Molecular Dynamics Simulations. J Chem Inf Model 52:2657–2669. https://doi.org/10.1021/ci3000453
4. Abraham MJ, Murtola T, Schulz R, et al (2015) GROMACS: High performance molecular simulations through multi-level parallelism from laptops to supercomputers. SoftwareX 1–2:19–25. https://doi.org/10.1016/j.softx.2015.06.001
5. Humphrey W, Dalke A, Schulten K (1996) VMD: Visual molecular dynamics. J Mol Graph 14:33–38. https://doi.org/10.1016/0263-7855(96)00018-5
6. Best RB, Zhu X, Shim J, et al (2012) Optimization of the Additive CHARMM All-Atom Protein Force Field Targeting Improved Sampling of the Backbone ϕ, ψ and Side-Chain χ_1 and χ_2 Dihedral Angles. J Chem Theory Comput 8:3257–3273. https://doi.org/10.1021/ct300400x
7. Huang J, MacKerell AD (2013) CHARMM36 all-atom additive protein force field: Validation based on comparison to NMR data. J Comput Chem 34:2135–2145. https://doi.org/10.1002/jcc.23354
8. Klauda JB, Venable RM, Freites JA, et al (2010) Update of the CHARMM All-Atom Additive Force Field for Lipids: Validation on Six Lipid Types. J Phys Chem B 114:7830–7843. https://doi.org/10.1021/jp101759q
9. Jorgensen WL, Chandrasekhar J, Madura JD, et al (1983) Comparison of simple potential functions for simulating liquid water. J Chem Phys 79:926–935. https://doi.org/10.1063/1.445869
10. Nosé S (1984) A molecular dynamics method for simulations in the canonical ensemble. Mol Phys 52:255–268. https://doi.org/10.1080/00268978400101201
11. Hoover WG (1985) Canonical dynamics: Equilibrium phase-space distributions. Phys Rev A (Coll Park) 31:1695–1697. https://doi.org/10.1103/PhysRevA.31.1695
12. Parrinello M, Rahman A (1980) Crystal Structure and Pair Potentials: A Molecular-Dynamics Study. Phys Rev Lett 45:1196–1199. https://doi.org/10.1103/PhysRevLett.45.1196

13. Nosé S, Klein ML (1983) Constant pressure molecular dynamics for molecular systems. Mol Phys 50:1055–1076. https://doi.org/10.1080/00268978300102851

14. Verlet L (1967) Computer "Experiments" on Classical Fluids. I. Thermodynamical Properties of Lennard-Jones Molecules. Physical Review 159:98–103. https://doi.org/10.1103/PhysRev.159.98

15. Páll S, Hess B (2013) A flexible algorithm for calculating pair interactions on SIMD architectures. Comput Phys Commun 184:2641–2650. https://doi.org/10.1016/j.cpc.2013.06.003

16. Darden T, York D, Pedersen L (1993) Particle mesh Ewald: An N ·log(N) method for Ewald sums in large systems. J Chem Phys 98:10089–10092. https://doi.org/10.1063/1.464397

17. Essmann U, Perera L, Berkowitz ML, et al (1995) A smooth particle mesh Ewald method. J Chem Phys 103:8577–8593. https://doi.org/10.1063/1.470117

18. Hess B (2008) P-LINCS: A Parallel Linear Constraint Solver for Molecular Simulation. J Chem Theory Comput 4:116–122. https://doi.org/10.1021/ct700200b

	Time Interval (ns)	Permeation Route Type
Run #1	855-858	1-6
	871-884	1-6
	936-950	1-6
Run #2	435-438	1-12
	468-472	1-12
	479-482	1-12
	638-641	1-6
	645-649	1-6
	649-653	intermediate
	685-688	intermediate
	709-712	1-6
	722-734	intermediate
	735-743	intermediate
	744-749	intermediate
	756-766	1-12
	775-782	1-6
	789-795	1-6

Table S1. Summary of all chloride translocation events in simulations with -500 mV transmembrane voltage

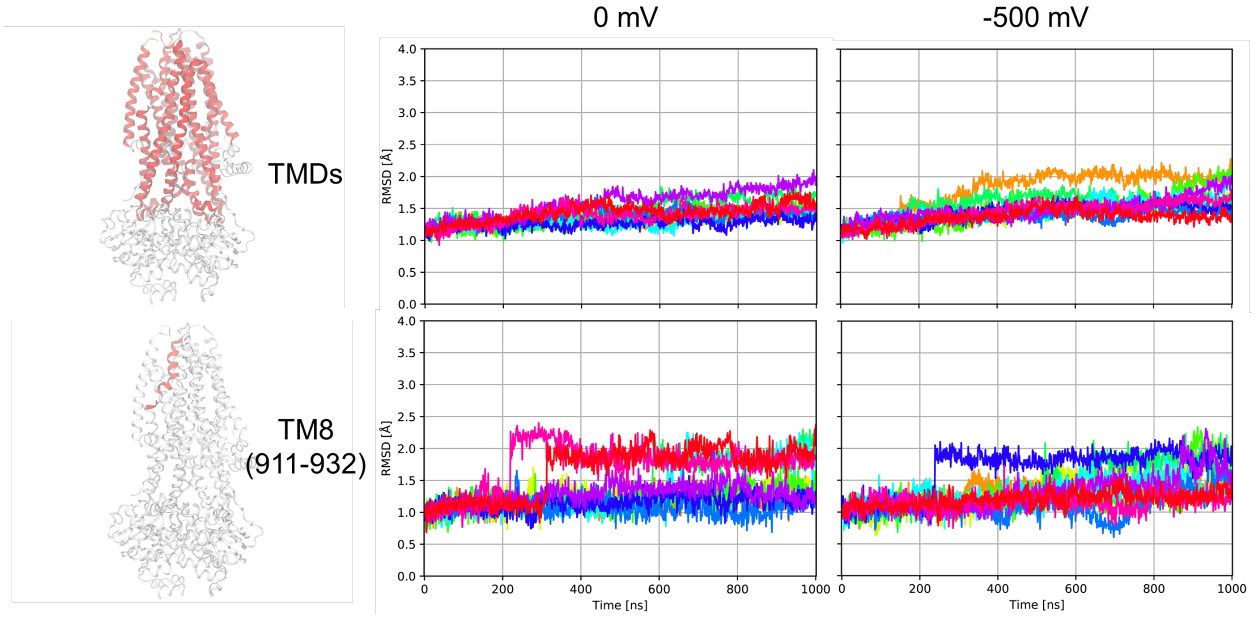

Fig. S1 RMSD of Cα atoms of transmembrane domains (TMDs) and of the extracellular segment of TM8 (residue 911-932) over time for all simulation trajectories. The positions of Cα atoms of TMDs in the experimentally determined structure (PDB: 6MSM) are used as the reference for structural alignments prior to RMSD calculations.

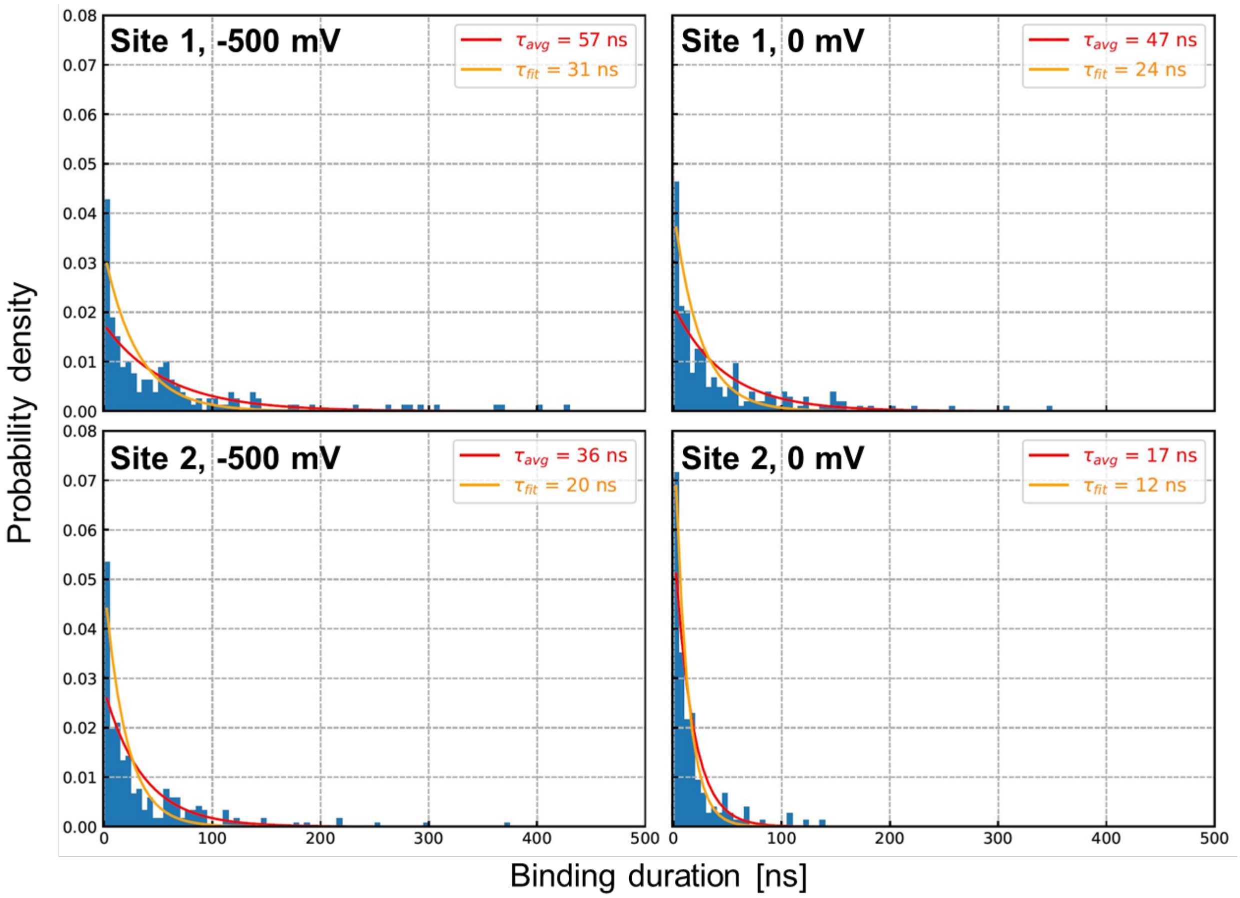

Fig. S2 Probability density histograms of Cl^- binding durations at sites 1 and site 2 (left) in the presence and (right) in the absence of transmembrane voltage. Estimates of τ values along with the theoretical probability density functions (red, orange) are shown (see Supplementary Methods).

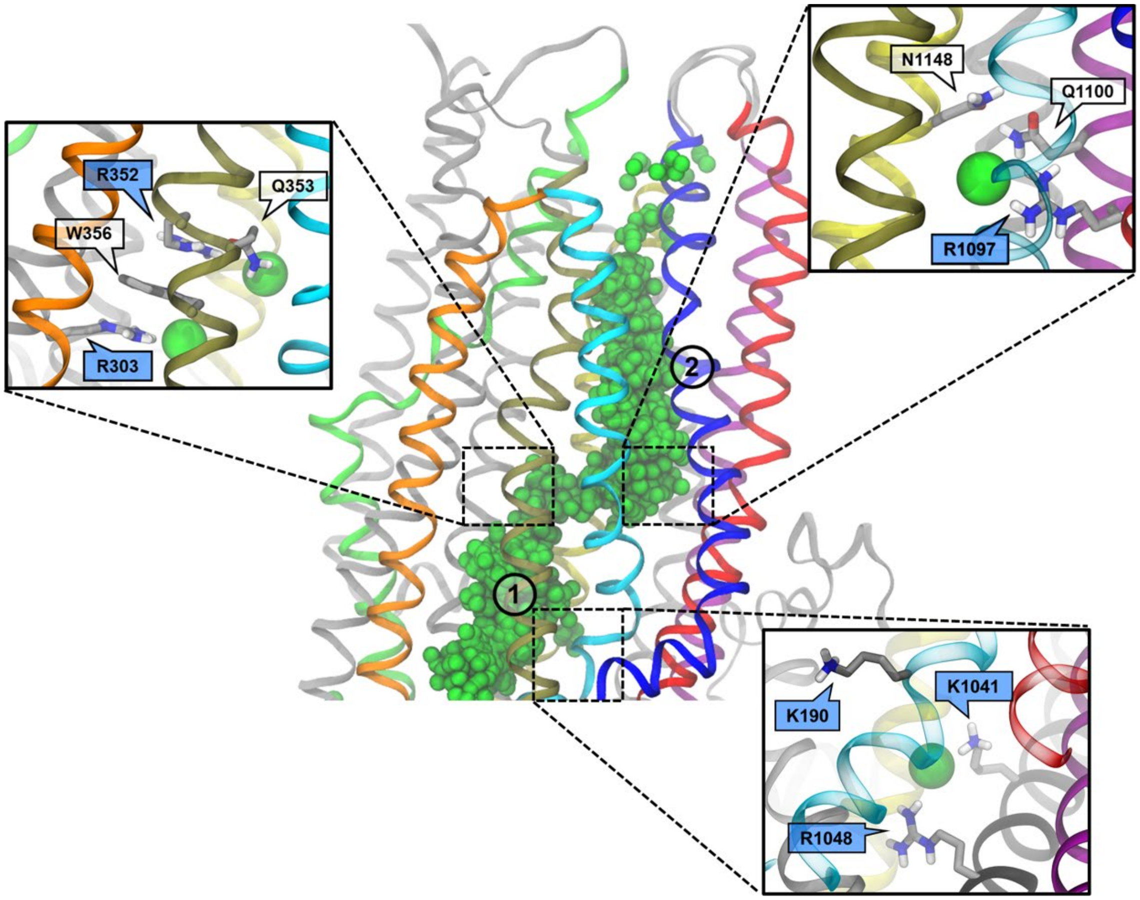

Fig. S3 Chloride pathway inside the inner vestibule of human CFTR with close-up views of other minor binding sites. Positions of major binding sites 1 and 2 are also indicated. Heavy atoms and polar hydrogen atoms of residue sidechains involved in direct interactions with Cl^- ions (green spheres) are shown in Licorice representation. These residues are labeled, with positively charged residues shaded in indigo. Helices are made translucent if they would otherwise obstruct the view of the binding sites. Selected TM helices are colour coded (blue: TM1; red: TM2; cyan: TM3; orange: TM4; tan: TM6; lime: TM8; purple: TM11; yellow: TM12).

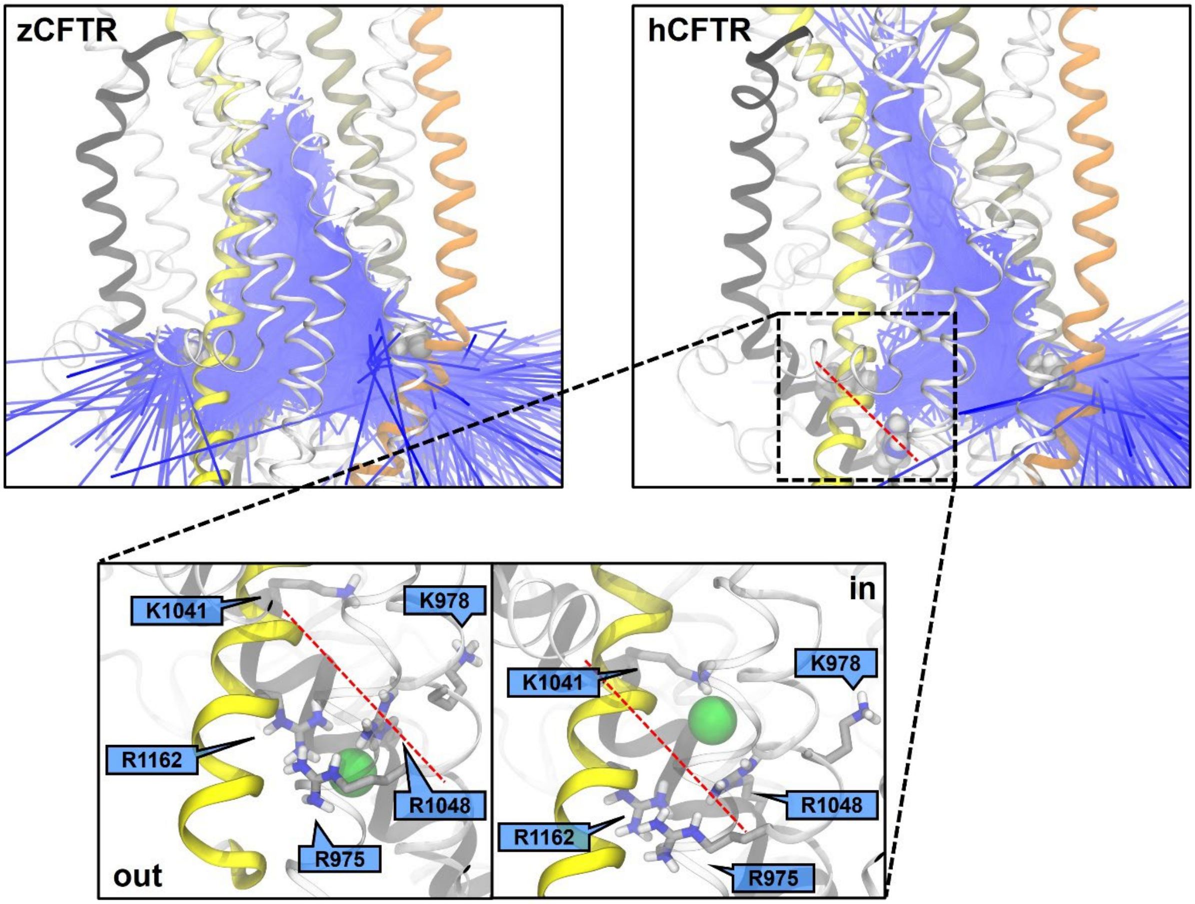

Fig. S4 Visualization of cytosolic entry pathways of Cl^- ions in simulations of (left) zebrafish CFTR (zCFTR, PDB: 5W81) and (right) human CFTR (hCFTR, PDB: 6MSM). Blue line segments connecting Cl^- ion positions at nanosecond intervals indicate their movements. Two lateral entrances (TM4-TM6 and TM10-TM12) were found in zCFTR, only one of which was observed in hCFTR (TM4-TM6). Insets: an auxiliary Cl^- binding site inside the pore of hCFTR is at the location corresponding to what would be the TM10-TM12 entrance ("in"). There is an adjacent cytosolic binding site for Cl^- ion outside the channel pore ("out"). Despite its close proximity to the site inside the pore, no instances of Cl^- entry into the pore was observed at this location (regardless of the presence of electric field). The dashed red line is used to delineate the two sites. Selected TM helices are colour coded (orange: TM4; tan: TM6; black: TM10; yellow: TM12).

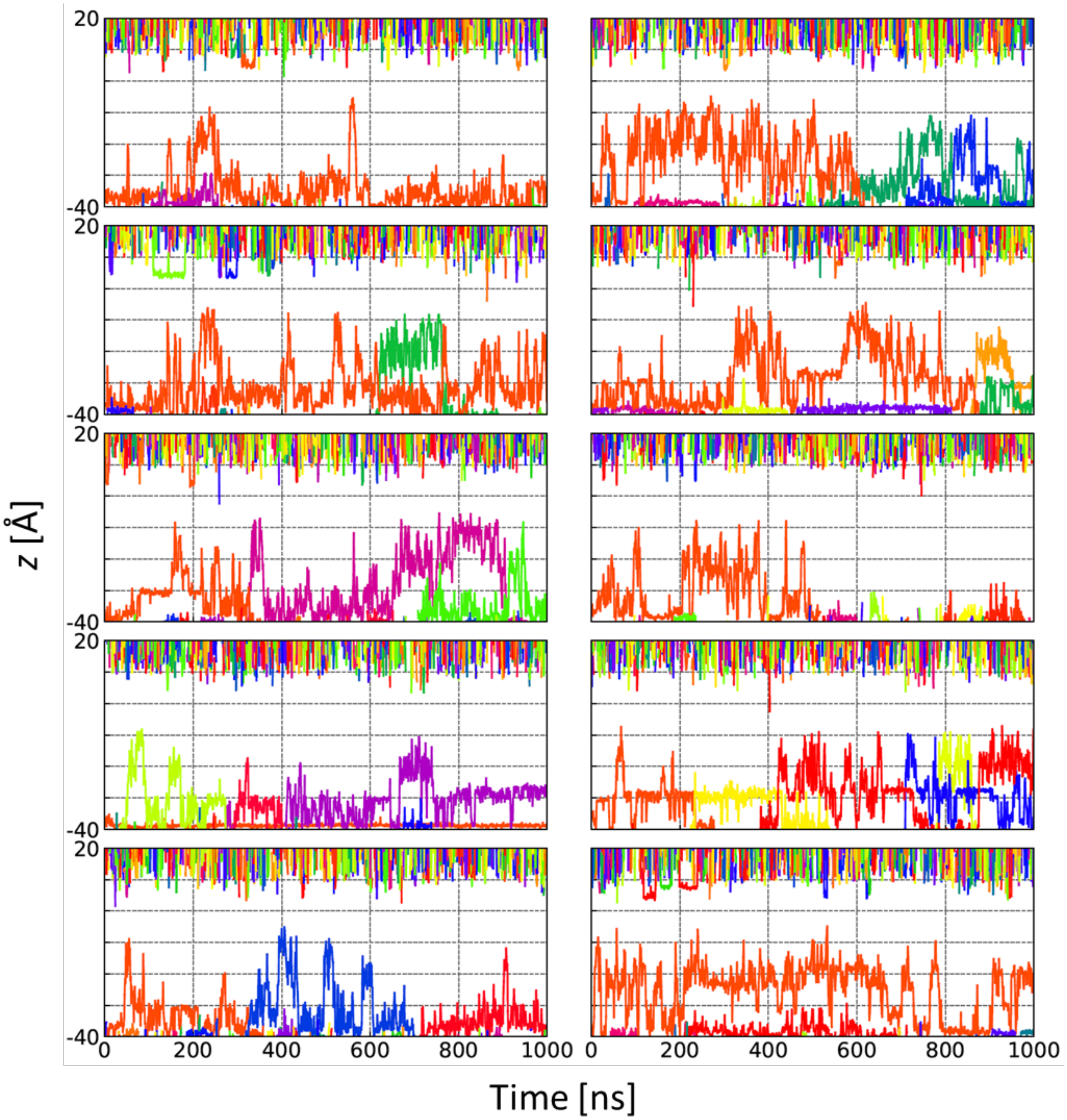

Fig. S5 Axial positions of Cl^- ions inside the CFTR channel over time for all ten simulation repeats conducted in the absence of transmembrane voltage.

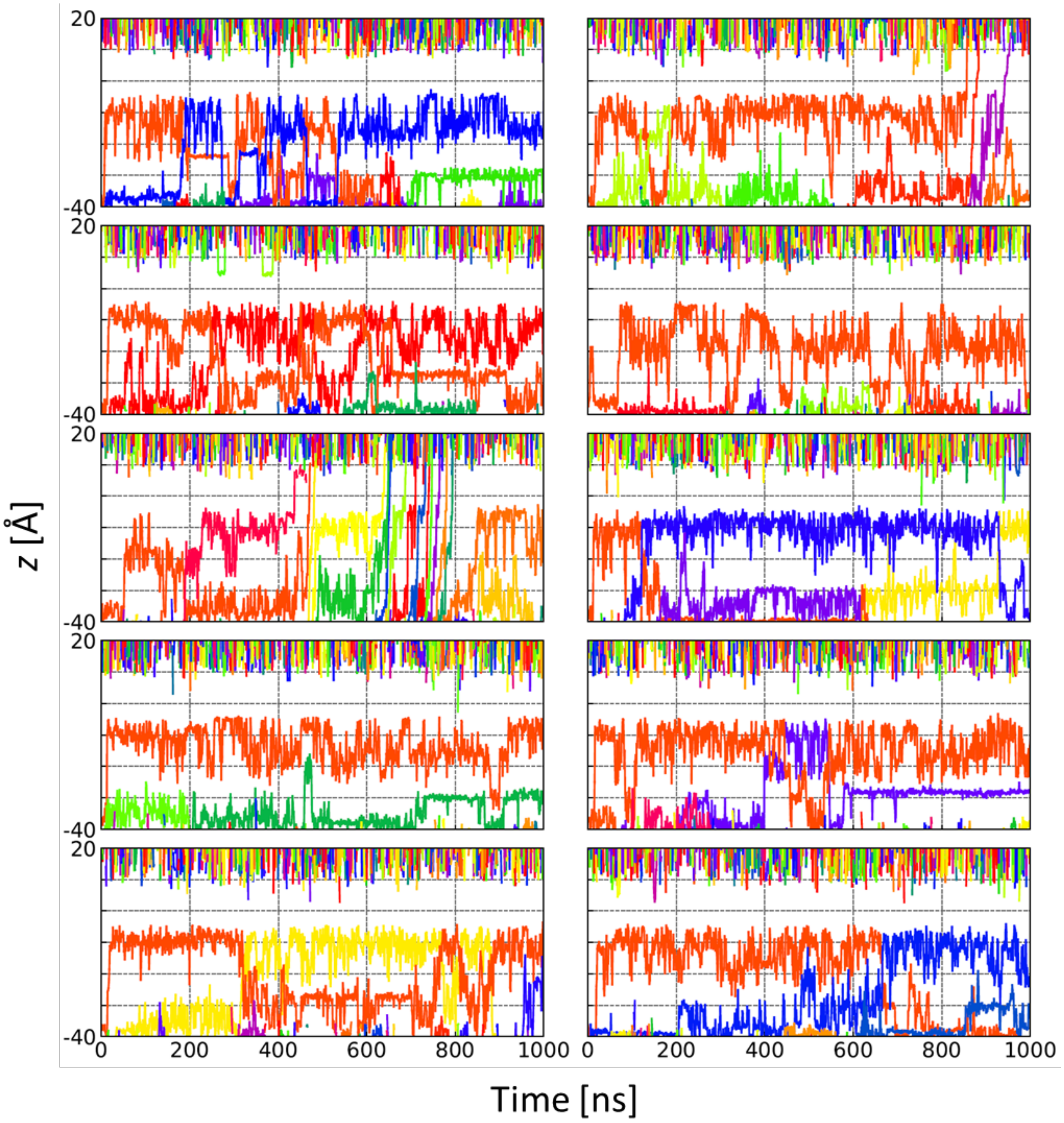

Fig. S6 Axial positions of Cl^- ions inside the CFTR channel over time for all ten simulation repeats conducted in the presence of transmembrane voltage.

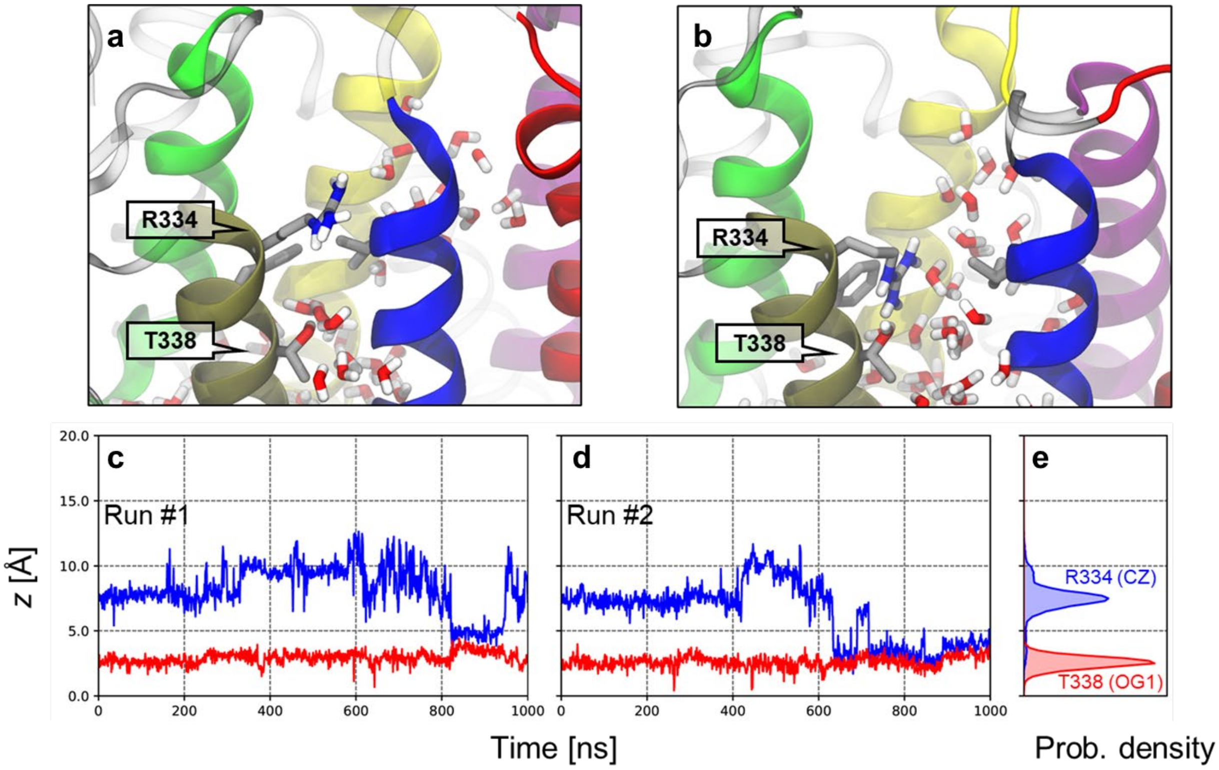

Fig. S7 Conformational fluctuations of protein side chains at the extracellular end of the pore. Top: visualizations of the extracellular region **(a)** before and **(b)** after permeation through the 1-6 or intermediate pathways. **(a)** Before permeation, the sidechain of R334 extends upwards, towards the extracellular space and away from the pore. **(b)** Following the opening of the gate, the R334 sidechain moves down into the hydrophobic region of the gate. **(c)-(d)** Time dependence of axial positions of R334 ζ-carbon atom (CZ) and T338 β-hydroxyl oxygen atom (OG1). The R334 sidechain plugs into the pore during Cl^- permeation through the 1-6 or intermediate pathways, thus explaining the ability of R334 to contact Cl^- ions before they reach T338. **(e)** Histograms of axial positions of R334 CZ atom and T338 OG1 atom in the presence of an external electric field.

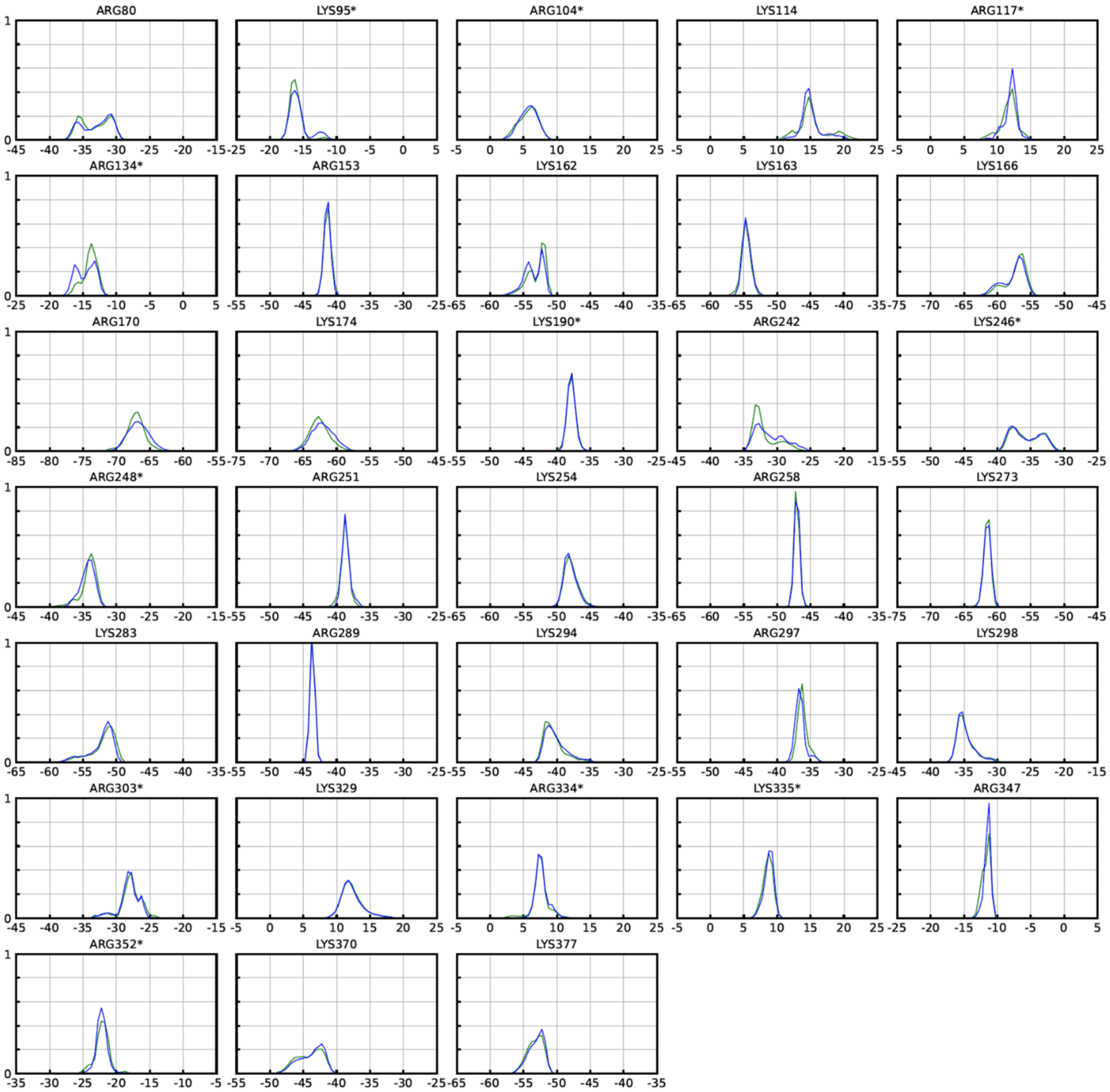

Fig. S8 Axial distributions of sidechains of lysine Nζ and arginine Cζ ratoms in hCFTR TM-domains from simulations in the presence (green) and absence (blue) of an external electric field (continued on the next page).

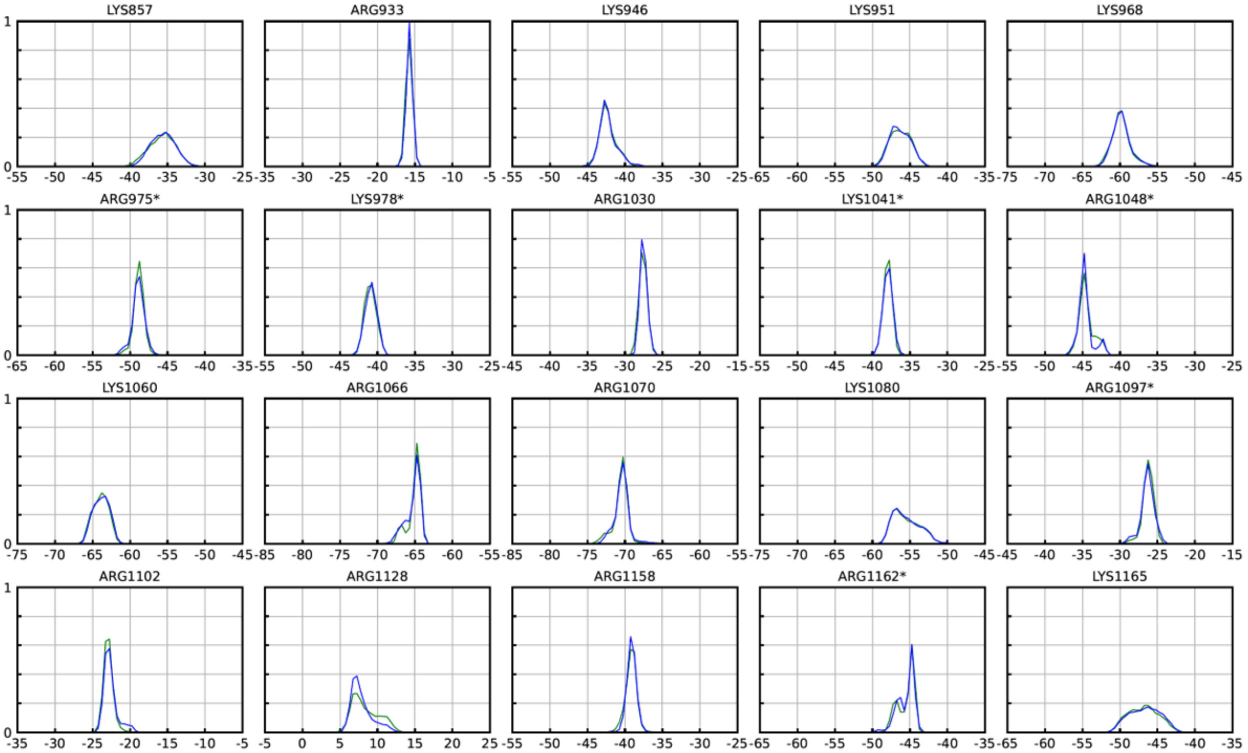
LYS857
ARG933
LYS946
LYS951
LYS968
ARG975*
LYS978*
ARG1030
LYS1041*
ARG1048*
LYS1060
ARG1066
ARG1070
LYS1080
ARG1097*
ARG1102
ARG1128
ARG1158
ARG1162*
LYS1165

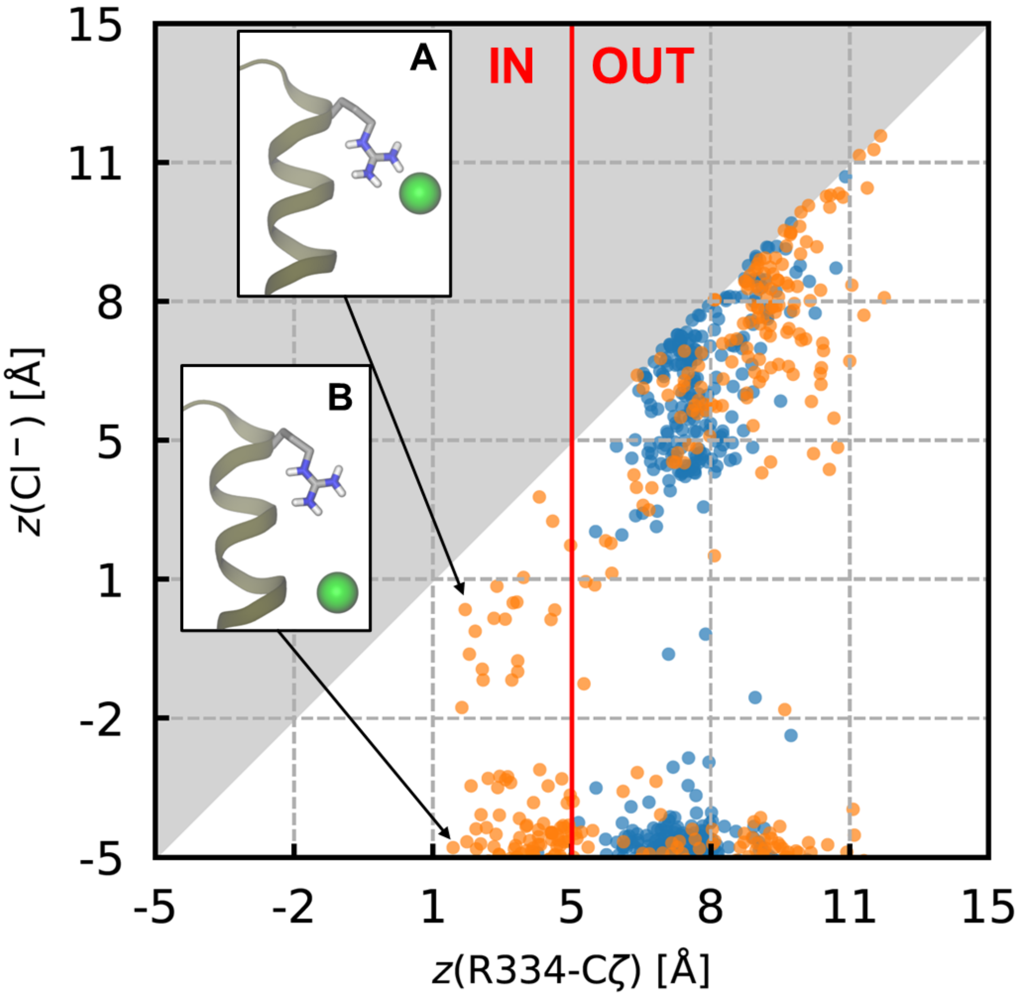

Fig. S9: Joint distribution of R334 and Cl^- in the pore bottleneck from simulations with voltage. The axial position of the approximate center of charge of the guanidinium group of R334, Cz, is compared to that of the closest Cl^- ion from below. Dot color distinguishes between simulations that reached the open state (orange) from those that did not (blue). The red line indicates the upper boundary of the bottleneck region and separates the dunked ("IN") and non-dunked ("OUT") conformations of the R334 sidechain. The shaded area corresponds to Cl^- above Cζ, which is excluded from the analysis. The dunked conformation of R334 [z(Cζ) < 5 Å] was only observed in the open state. In the dunked state, R334 can form a contact ion pair with Cl^- in the bottleneck (inset A), although it is often seen without Cl^- in close proximity (inset B).

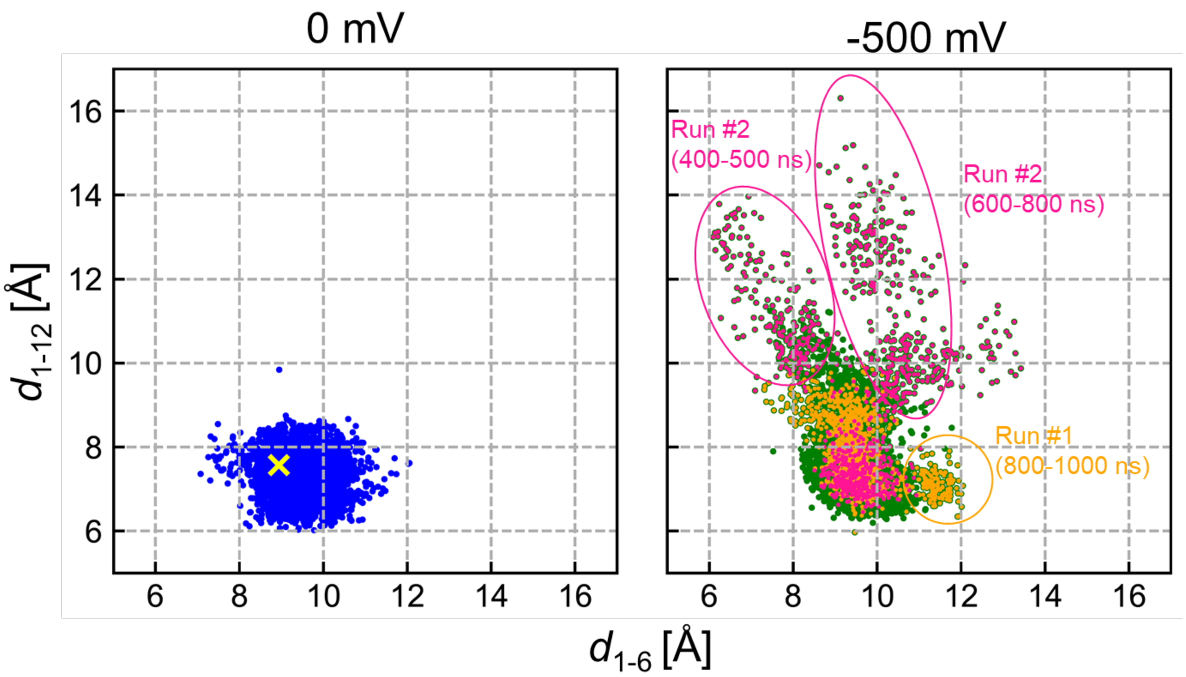

Fig. S10 Scatter plots of TM1-TM12 distance vs TM1-TM6 distance at the extracellular end, based on positions of Cα of residues in Fig. 8a. Left: simulations without voltage. The position corresponding to the cryo-EM structure (PDB: 6MSM) is indicated as a yellow “x”. Right: simulations with voltage. Scatter points corresponding to run #1 (orange) and run #2 (pink) are indicated separately. Time intervals of runs #1 and #2 in which chloride permeation occurred are circled.

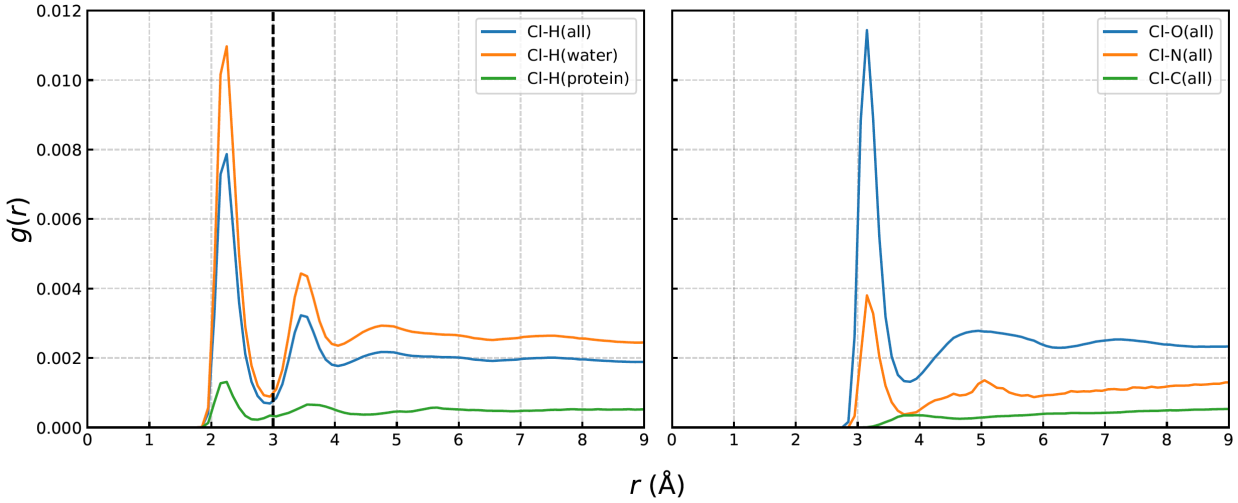

Fig. S11 Atomic radial pair distribution functions *g*(*r*) between chloride ions and protein or solvent atoms (*r*: distance from chloride ion). Cut-off radius of 3 Å (left: black dashed line) was used in determining the occurrence of ion solvation or coordination (with hydrogen atoms taken into account; see Methods).
